# Notch3 contributes to T-cell leukemia growth via regulation of the unfolded protein response

**DOI:** 10.1038/s41389-020-00279-7

**Published:** 2020-10-18

**Authors:** Maria Valeria Giuli, Giulia Diluvio, Eugenia Giuliani, Giulia Franciosa, Laura Di Magno, Maria Gemma Pignataro, Luca Tottone, Carmine Nicoletti, Zein Mersini Besharat, Giovanna Peruzzi, Maria Pelullo, Rocco Palermo, Gianluca Canettieri, Claudio Talora, Giulia d’Amati, Diana Bellavia, Isabella Screpanti, Saula Checquolo

**Affiliations:** 1 Laboratory of Molecular Pathology, Department of Molecular Medicine, Sapienza University, Rome, Italy; 2 Sylvester Comprehensive Cancer Center, Miller School of Medicine, University of Miami, Miami, FL USA; 3 Center for Life Nano Science@Sapienza, Istituto Italiano di Tecnologia, Rome, Italy; 4 Novo Nordisk Foundation Center for Protein Research, University of Copenaghen, Copenaghen, Denmark; 5 Department of Radiological, Oncological and Pathological Sciences, Sapienza University, Rome, Italy; 6 Rutgers Cancer Institute of New Jersey, Rutgers University, New Brunswick, NJ USA; 7 Unit of Histology and Medical Embryology, Department of Anatomy, Histology, Forensic Medicine and Orthopaedics, Sapienza University, Rome, Italy; 8 Department of Medico-Surgical Sciences and Biotechnology, Sapienza University, Latina, Italy

**Keywords:** Cell biology, Leukaemia

## Abstract

Unfolded protein response (UPR) is a conserved adaptive response that tries to restore protein homeostasis after endoplasmic reticulum (ER) stress. Recent studies highlighted the role of UPR in acute leukemias and UPR targeting has been suggested as a therapeutic approach. Aberrant Notch signaling is a common feature of T-cell acute lymphoblastic leukemia (T-ALL), as downregulation of Notch activity negatively affects T-ALL cell survival, leading to the employment of Notch inhibitors in T-ALL therapy. Here we demonstrate that Notch3 is able to sustain UPR in T-ALL cells, as Notch3 silencing favored a Bip-dependent IRE1α inactivation under ER stress conditions, leading to increased apoptosis via upregulation of the ER stress cell death mediator CHOP. By using *Juglone*, a naturally occurring naphthoquinone acting as an anticancer agent, to decrease Notch3 expression and induce ER stress, we observed an increased ER stress-associated apoptosis. Altogether our results suggest that Notch3 inhibition may prevent leukemia cells from engaging a functional UPR needed to compensate the *Juglone*-mediated ER proteotoxic stress. Notably, in vivo administration of *Juglone* to human T-ALL xenotransplant models significantly reduced tumor growth, finally fostering the exploitation of *Juglone*-dependent Notch3 inhibition to perturb the ER stress/UPR signaling in Notch3-dependent T-ALL subsets.

## Introduction

T-cell acute lymphoblastic leukemia (T-ALL) is an aggressive hematologic tumor resulting from the malignant transformation of T-cell progenitors. T-ALL accounts for ~15% and 25% of ALLs seen in children and adults, respectively^1^. Notch receptors have been implicated as oncogenic drivers in a number of different human cancers, including T-ALL, which shows increased Notch1 activity in about 60% of cases, due to activating Notch1 mutations or alterations in the FBXW7 gene^2,3^. By screening primary T-ALL tumors and orthotopic patient-derived xenograft models, activating mutations of Notch3 have been recently identified, also detectable in the absence of an activated Notch1^4^. Since constitutive activation of the Notch signaling pathway confers to the leukemic cells a strong growth advantage, the Notch therapeutic targeting has assumed a considerable clinical relevance, especially for patients refractory to chemotherapy^5^. However, the gamma-secretase inhibitors (GSIs) treatment, which blocks cleavage of Notch receptors, exhibits significant gastrointestinal toxicity, mainly due to the simultaneous inhibition of Notch1 and Notch2 signaling in gut epithelial stem cells^6^.

In this scenario, the endoplasmic reticulum (ER) stress/unfolded protein response (UPR) pathway is gaining increasing recognition as a key targetable pathway in acute leukemias^7^. Tumor cells are often exposed to different stimuli that cause ER stress: adaptation to stress and re-establishment of ER homeostasis is achieved by activation of an integrated signal transduction pathway called UPR^8^. Three major branches of the UPR have been identified: IRE1α (inositol-requiring enzyme 1 alpha), PERK (double-stranded RNA-activated protein kinase (PRK)-like ER kinase), and ATF6 (activating transcription factor 6). Under unstressed conditions, the stress sensors are maintained inactive through binding to the ER chaperone GRP78/Bip. After ER stress induction, GRP78/Bip dissociates from UPR sensors, thereby leading to their activation. Importantly, by integrating transcriptional and translational responses, UPR makes life/death decisions for the cell and the final outcome of ER stress is either recovery and cell survival or apoptosis, depending on the severity and duration of ER stress^8^. Targeting the UPR for cancer treatment is considered a promising approach, as the UPR appears to be activated in a variety of human tumors. Interestingly, the downregulation of UPR signaling was shown to drive apoptotic cell death in T-ALL^9,10^. However, it remains to be fully elucidated how different oncogenes are able to influence the UPR autonomously or through interaction with the ER sensors, raising the possibility to identify a new therapeutic opportunity for T-ALL-bearing patients.

Here, we revealed an unknown role of Notch3 protein in sustaining the activation of the UPR pathway through its involvement in the ER stress/UPR signaling network regulation. By using a canonical ER stress inducer, Tunicamycin, we observed that the combined downregulation of Notch3 protein expression (but not of Notch1) was able to favor the ER stress-associated IRE1α ubiquitination and inactivation, in a GRP78/Bip-dependent manner. This event prevented leukemic cells from engaging a functional UPR required to counteract the ER-mediated proteotoxic stress, finally leading to ER-associated pro-apoptotic events, represented by increased levels of the ER stress cell death mediator CHOP.

In order to evaluate in vivo the potential anti-leukemic effects derived from the previously reported combination of Notch3 downregulation under ER stress conditions, in this study we used the *Juglone* (5-hydroxy-1,4-naphthoquinone), a naturally occurring naphthoquinone derived from the *Juglans mandshruica Maxim*, that has shown strong activity against cancer cells, including human leukemia^11,12^. Interestingly, we demonstrated that *Juglone* treatment resulted in the Notch3 downregulation, IRE1α ubiquitination/inactivation, and amplification of ER-associated pro-apoptotic events. Furthermore, we also observed that *Juglone* was able to induce Notch3 downmodulation and CHOP induction in vivo, finally exerting anti-leukemia growth in a human T-ALL xenograft mouse model. Taken together, our findings provide a rationale for the use of Notch3 inhibition and/or *Juglone*-based therapy protocols in the treatment of a Notch3-dependent subset(s) of T-ALLs.

## Materials and methods

### Cell culture and treatments

Murine N3-232T^13^ and human leukemic cells (TALL-1, Jurkat, Ke37, KOPKT1, DND41, Molt3, P12-lchikawa, and SIL-ALL)^14,15^ were maintained as described elsewhere and all are mycoplasma-free. Cells were treated with different doses (as indicated in some Figures) or fixed 2.5 μM of *Juglone* (Calbiochem, San Diego, CA, USA, Cat#420120), 2.5 μM Thapsigargin (Sigma, St Louis, MO, USA, Cat#T9033) or 5μM Tunicamycin (Sigma, Cat#T7765) for the times indicated, according to their datasheets’ instructions. In some cases, cells were treated with 30 μM MG132 (Z-Leu-Leu-Leu-al; Sigma, Cat#C2211) for 6 h before harvesting. In some experiments (IP assays), cells were treated with *Juglone* for 6–8 h at maximum, in order to maintain the cell viability over 80% and to avoid an important increase in cell death before analysis.

For survival analysis, cells were harvested at different time points and counted by using a Trypan blue assay. To evaluate compound synergy, we used the Excess-over-Bliss (EOB) score for a selected pair of concentrations of siRNA-N3 (200 nM) and *Juglone* (2.5μM). EOB value indicates the difference between the observed and predicted inhibition of the compound combination^16^. For EOB < 0, there is an antagonistic effect; for EOB = 0 there is an addictive effect; for EOB > 0, there is a synergistic effect.

Primary T-ALL cells (PDTALLs) included in the present studies were kindly provided by Dr. Indraccolo’s lab^17^. We selected a group of PDTALL available samples based on their Notch1 expression (wild-type and mutated) and we screened them for the expression of Notch3. PDTALL cells were grown in vitro for 24 h in MEM alpha medium (Life Technologies, Paisley, UK), supplemented with 10% fetal calf serum (FCS), 10% human heat-inactivated AB+ serum, 1% penicillin/streptomycin, 1% Glutamax (all from Life Technologies), human IL7 (10 ng/ml), human SCF (50 ng/ml), human FLT3-ligand (20 ng/ml) (all from Peprotech, London, UK) and insulin (20 nM) (Sigma-Aldrich, St Louis, MO). One day later, T-ALL cells were seeded (0.25 * 10^6^/well) and treated for 24 h with different doses (as indicated in the Figure) or fixed 2.5 μM *Juglone* before cell harvesting and western blot or flow cytometric analysis.

### Flow cytometric analysis

To determine the extent of apoptosis induction after drug treatment, flow cytometric analysis of Annexin V (BD Pharmigen, San Diego, CA, USA, Cat#550474)/propidium iodide (PI) (BD Pharmigen, Cat#556463) stained samples was performed, as described elsewhere^18^. Then, samples were analyzed on a FACS-Calibur with CellQuest software (BD-Biosciences, San Jose, CA, USA).

### RNA extraction, RT-PCR and qRT-PCR, and Notch knockdown

Total RNA extraction and reverse transcription (RT-PCR) were previously described^19,20^. The expression levels of GRP78/Bip, CHOP, and GAPDH mRNAs were determined by SYBR Green quantitative real-time RT-PCR (qRT-PCR) performed on cDNA according to the manufacturer’s instructions (Applied Biosystems, Life Technologies Brand, Carlsbad, CA, USA) and using the ABI Prism 7900HT (Applied Biosystems). Data were analyzed by the ΔΔCt method and GAPDH was used to normalize the expression levels of mRNA^21^. RT-PCR for XBP1 mRNA splicing analysis and β-actin was performed using Taq Gold polymerase. The amplicons were resolved using a 2% agarose gel. The details of the primers for each gene are given in Supplementary Table S1. Measurements were performed in technical triplicates and figures show the average ± SD from an appropriate number of experiments (at least three biological replicates). Cells were silenced for Notch3 and Notch1 genes as previously described^22^, by using two different sequences (#1 and #2) for each human gene: from Santa Cruz Biotechnology (Santa Cruz, Dallas, TX, USA), siN3 #1 (sc-37135), siN1 #1 (sc-36095), and corresponding control scrambled siRNAs #1 (sc-37007); from ThermoFisher Scientific (Waltham, MA, USA), siN3 #2 (106100), siN1 #2 (S9634), and corresponding *Silencer*™ Negative Control siRNAs #2 (AM4611).

### Western blot, immunoprecipitation assay, and antibodies

Protein extracts preparation, immunoprecipitation and immunoblotting assays were performed as previously described^23,24^. Antibodies: from Cell Signalling (Danvers, MA, USA), anti-Notch3 (Cat#2889); anti-Notch1 (Cat#3608); anti-GRP78/Bip (Cat#3177); anti-IRE1α (Cat#3294); anti-CHOP (Cat#2895); anti-PARP (Cat#9542); anti-PERK (Cat#5683P); from Santa Cruz Biotechnology (Dallas, TX, USA), anti-Lamin B M20 (Cat#sc-6217), anti-ubiquitin (P4D1; Cat#sc-8017); anti-ATF-6 (H-280; Cat#sc-22799); from Sigma-Aldrich (Saint Louis, MO, USA), anti-β-actin (Cat#A5316). The anti-N3_EC_ (5E1) antibody was kindly provided by Professor A Joutel, as previosly described^13^.

### Immunohistochemistry

Tissue samples were fixed and paraffinized as described^25^. The 4 μm thick sections were prepared from paraffin-embedded tissues and immunostained with anti-CD45 (X16/99, Novocastra, Leica biosystems, Newcastle, UK, Cat# NCL-L-LCA), anti-Notch3 (Santa Cruz Biotechnology, Dallas, TX, USA, Cat#5539) or anti-CHOP (Cell Signalling, Danvers, MA, USA, Cat#2895) antibodies. After washes, secondary biotinylated antibodies were applied. Binding of antibodies was detected with the Mouse to Mouse HRP (DAB) Staining System (Scytek Laboratories, Inc., Logan, UT, USA) according to the manufacturer’s protocol. The analysis was conducted blindly.

### Statistical analysis

Results are expressed as mean ± SD. All statistical tests were carried out by using GraphPad Prism version 7.0 (GraphPad Software, San Diego, California, USA). A comparison analysis between two groups was carried out by using a two-tailed Student’s unpaired *t*-test. For multiple comparisons of groups, a one-way analysis of variance was used. Among the groups that we have statistically compared we observed similar variance. Multiple comparison analysis was carried out by one-way ANOVA followed by Sidak’s or Dunnett’s post-hoc tests. Differences were considered significant for *P* < 0.05. Values significance: *P ≤ 0.05; **P < 0.01; ***P < 0.001^26^. In some cases, the index Pearson *R* is also indicated to express a possible linear relation between paired samples. All data shown are representative of at least three independent experiments and the repeat number was increased according to effect size or sample variation. We estimated the sample size considering the variation and mean of the samples. No statistical method was used to predetermine the sample size. No animals or samples were excluded from any analysis.

## Results

### Notch3 modulation influences T-ALL cells survival in response to ER stress induction

**Fig. 1:**
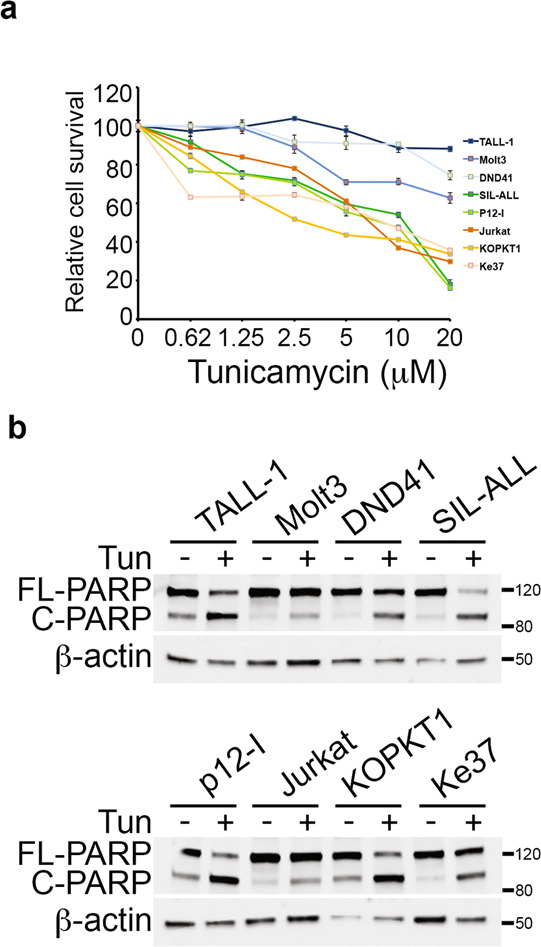
Differential T-ALL cells survival in response to ER stress induction. **a** Cell survival of T-ALL cells treated with increasing doses of Tunicamycin for 24 h. Results are shown as the means average deviations of at least three independent experiments and adjusted *P*-values of all comparison pairs assessed with ANOVA/Dunnett’s post-hoc test are shown in the lower panel (i.e., ns, not significant; **P ≤ 0.01; ***P ≤ 0.001). For all the untreated cells (0 μM), excepted for Molt3 (siN3 #1 and siN1 #2) and DND41 (siN3 #2), no significant differences are observed (*P*-value: *P* = 0.05). **b** Representative western blot of cleaved and full-length form of PARP (C-PARP and FL-PARP, respectively) in T-ALL cells (TALL-1; Molt3; DND41; SIL-ALL; p12-I; Jurkat; KOPKT1; Ke37) after 20 μM Tunicamycin treatment shown in **a**. β-actin was used as loading control. p12-I p12-Ichikawa cells.

**Fig. 2:**
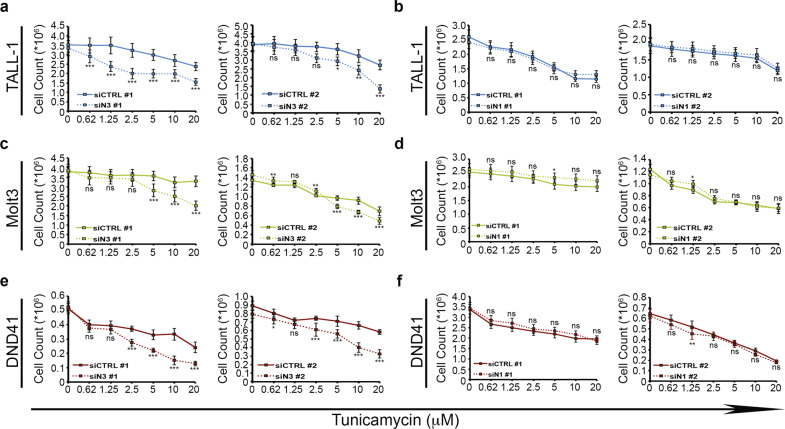
Notch3 silencing favors the response to ER stress in T-ALL. **a**–**f** Cell survival of Notch3-silenced (siN3) or Notch1-silenced (siN1) TALL-1 (**a**, **b**), Molt3 (**c**, **d**) or DND41 (**e**, **f**) cells, treated with increasing doses of Tunicamycin compared with control cells CTRL for 24 h. For both Notch3 and Notch1 silencing experiments, left and right panels showed the use of the first (#1) and the second (#2) sequence of siRNA, respectively. Results are shown as the means average deviations of three independent experiments and adjusted *P*-values of all comparison pairs (CTRL vs siN3/N1 at each single dose) assessed with ANOVA/Sidak’s post-hoc test are shown (i.e., ns not significant; *P ≤ 0.05; **P ≤ 0.01; ***P ≤ 0.001).

To assess the involvement of Notch proteins in the ER stress/UPR signaling network in T-ALL, ER stress induction by Tunicamycin treatment was performed in different human T-ALL cell lines, all constitutively expressing Notch1 and Notch3 at various levels (Supplementary Fig. S1a), and their survival was evaluated (Fig. 1a). Tunicamycin decreased the proportion of viable cells by increasing cell death, as we observed an increase in the pro-apoptotic cleaved form of poly ADPribose polymerase PARP (C-PARP) in all T-ALLs analyzed (Fig. 1b). Interestingly, all the leukemic cells expressing higher levels of Notch3 (TALL-1, DND41, Molt3) appeared more resistant to increasing doses of Tunicamycin while Notch3^low^ (Jurkat and KOPKT1) and Notch3^neg^ (Ke37) cells did not (Fig. 1a), independently of Notch1 expression levels and its mutational *status* (Supplementary Fig. S1a). In agreement with this, downregulation of Notch3 expression (but not of Notch1) alters Tunicamycin response: indeed, Notch3-silenced cells (siN3) displayed a significant decreased cell survival with respect to control cells (siCTR) (Fig. 2a, c, e and Supplementary Fig. S2a, c, e) while the Notch1-silenced cells (siN1) did not (Fig. 2b, d, f and Supplementary Fig. S2b, d, f). All together our observations suggested a potential novel role of Notch3 in sustaining the response to ER stress induction in T-ALLs.

### Notch3 modulation affects ER stress/UPR signaling by regulating IRE1α protein expression

**Fig. 3:**
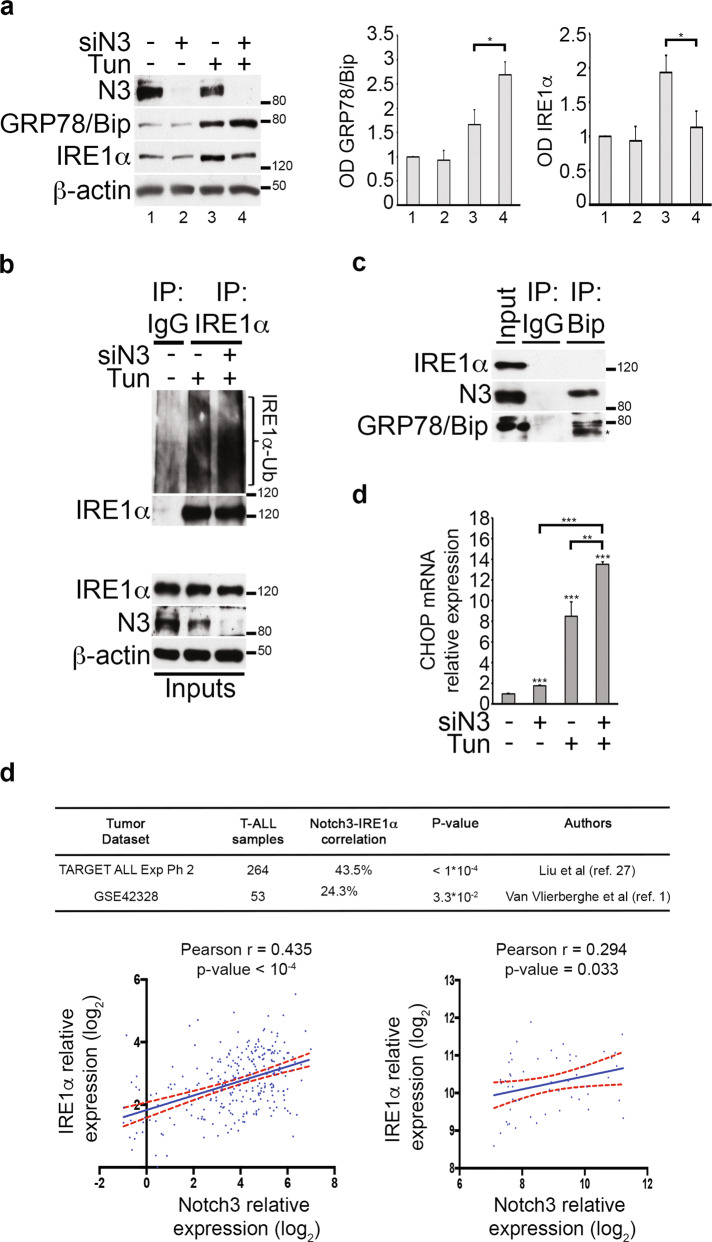
Notch3 modulation in Tunicamycin-treated TALL-1 cells induces UPR defects by decreasing IRE1α expression. **a** Western blot analysis of Notch3-silenced (siN3) TALL-1 cells treated with Tunicamycin (Tun) for 24 h showed that drugs synergized in increasing GRP78/Bip expression but not in IRE1α expression. Right panels, optical densitometry (OD) of GRP78/Bip and IRE1α protein expression levels analyzed in all the experiments performed (at least 3 biological replicates), thus including the *P*-values, calculated using Student’s *t*-test (i.e., *P ≤ 0.05). The number of the lanes correspond to: 1: siCTR; 2: siN3; 3: Tun; 4. siN3+Tun. **b** Control or anti-IRE1α immunoprecipates from the same cells used in **a** were probed with an anti-Ubiquitin (Ub) and anti-IRE1α antibodies to detect the Ubiquitination status of IRE1α (IRE1α-Ub) and IRE1α immunoprecipitated protein levels, respectively. Proteasomal inhibition with MG132 was used. Anti-N3 and anti-IRE1α antibodies were used to detect the total levels of Notch3 and IRE1α proteins, respectively. In both panels **a** and **b** the anti–β-actin antibody was used as a loading control. **c** Control or anti-GRP78/Bip (Bip) immunoprecipates from TALL-1 cells were subjected to western blot and probed with anti-IRE1α and anti-N3 antibodies to analyze the endogenous GRP78/Bip-IRE1α and GRP78/Bip-Notch3 interactions, respectively. The blot with anti-GRP78/Bip antibody was used to detect the GRP78/Bip immunoprecipitated protein levels. The input lanes indicated in all the western blot of the panels **b** and **c** show 5% of total lysate. * non-specific band?. All data are representative of at least three independent experiments, each in triplicate. **d** Relative CHOP mRNA expression derived from TALL-1 cells described in **a**. Results are shown as the means average deviations of at least three separate experiments and *P*-values were calculated using Student’s *t*-test (i.e., **P ≤ 0.01). **e** Upper panel: summary of the Notch3-IRE1α gene expression levels correlation obtained by an in silico analysis from two T-ALL data set (TARGET ALL Expansion Phase 2 and GSE42328). Lower panels: representative graphs showing correlation between Notch3 and IRE1α gene expression levels from (left) TARGET ALL Expansion Phase 2 data set in a cohort of 264 T-ALL patients and (right) GSE42328 data set in a cohort of 53 T-ALL patients. In both graphs, each dot corresponds to one patient and the expression value of Notch3 (*X* axis) and IRE1α (*Y* axis) is given in log2 scale after normalizing data with justRMA algorithm normalization. The index Pearson R indicated expresses the linear relation between paired samples and *P*-values were calculated using Student’s *t*-test, as described in the “Material and methods” section.

Digging deeper into the molecular mechanism underlying the possible Notch3-UPR cross-talk in T-ALL cells, we focused our in vitro studies on the Notch3-overexpressing TALL-1 leukemic cells, which display constitutive activation of Notch3^27^, while neither bearing Notch1-activating mutations nor displaying Notch1 activation^28^. The specific Notch3 downregulation induced during ER stress conditions (siN3 + Tun) was able to both enhance GRP78/Bip expression, a known marker of UPR activation^29^, and to attenuate the Tunicamycin-induced increase of IRE1α expression (Fig. 3a) while the other UPR sensors expression (i.e., ATF6 cleavage and PERK) remained unaffected (Supplementary Fig. S3a). By the consequence, we focused our attention on the IRE1α pathway, known to be the most conserved branch of the UPR^30^. In keeping with these data, by using the same experimental conditions, we observed the increase of endogenous IRE1α ubiquitination levels (Fig. 3b), possibly occurring through a known mechanism of IRE1α protein regulation, GRP78/Bip-dependent, previously described only under ER stress conditions^31^, when high levels of GRP78/Bip were induced (Fig. 3a). These observations suggested a direct involvement of Notch3 in the regulation of the ER stress/UPR markers expression but only within an ER stress-microenvironment, as the absence of Notch3 alone (siN3) did not correlate with any change in GRP78/Bip and IRE1α levels (Fig. 3a and Supplementary Fig. S4a, b). In addition, the ER stress-activated XBP1 splicing, which measures the IRE1α-dependent endoribonuclease activity during ER stress, remained unchanged (Supplementary Fig. S4c).

To evaluate the mechanistic relationship between Notch3 and the above-mentioned UPR sensors, we further analyzed its role with respect to the known GRP78/Bip-IRE1α cross-talk. As shown in Fig. 3c, we observed an unexpected endogenous Notch3-GRP78/Bip association in basal conditions and the absence of the known endogenous GRP78/Bip-IRE1α interaction, normally occurring in unstressed cells. This observation suggests the hypothesis of a Notch3-dependent GRP78/Bip sequestering which may justify the presence of an active ER stress/UPR machinery in TALL-1 cells, finally responsible for sustaining their survival. Interestingly, we observed the Notch3-GRP78/Bip association also in Jurkat cells which express wild-type Notch3 protein (Supplementary Fig. S5), thus excluding the possibility of an exclusive role of the mutant Notch3 in TALL-1 cells, probably more prone to misfolding and therefore needing the chaperone GRP78/Bip for the proper folding.

Our findings demonstrated that Notch3 downmodulation under ER stress conditions correlates with an UPR defect by favoring the GRP78/Bip-overexpression effects upon IRE1α protein level, which resulted in IRE1α downregulation (Fig. 3). Even if we did not observe any significant changes in the other UPR sensors expression (Supplementary Fig. S3), the Notch3-dependent alteration of IRE1α expression appears sufficient to influence the ER stress/UPR balance towards the pro-apoptotic mode, as we observed a combined significant increase in the mRNA expression of CHOP, a pro-apoptotic transcription factor induced by severe or prolonged ER stress conditions (Fig. 3d).

Notably, an in silico analysis of the Notch3 and IRE1α gene expression levels in two cohorts of T-ALL-bearing patients, collectively consisting of 317 individuals^1,32^ (Fig. 3e), highlighted a significant positive correlation between Notch3 and IRE1α gene expression levels, thus reinforcing the possible relationship between Notch3 and IRE1α proteins in T-ALL context.

### *Juglone* acts simultaneously by inducing ER stress and downregulating Notch3 in TALL-1 cells

In order to evaluate the potential anti-leukemic effects of the Notch3 downregulation under ER stress conditions previously observed (Figs. 2 and 3, siN3+Tun) also in in vivo studies, we chose the natural compound *Juglone* for the subsequent experiments, since several literature’s data^11,33–35^, including ours^36^, showed its ability to induce different molecular mechanisms potentially resulting in the simultaneous ER stress induction and Notch3 blocking.

**Fig. 4:**
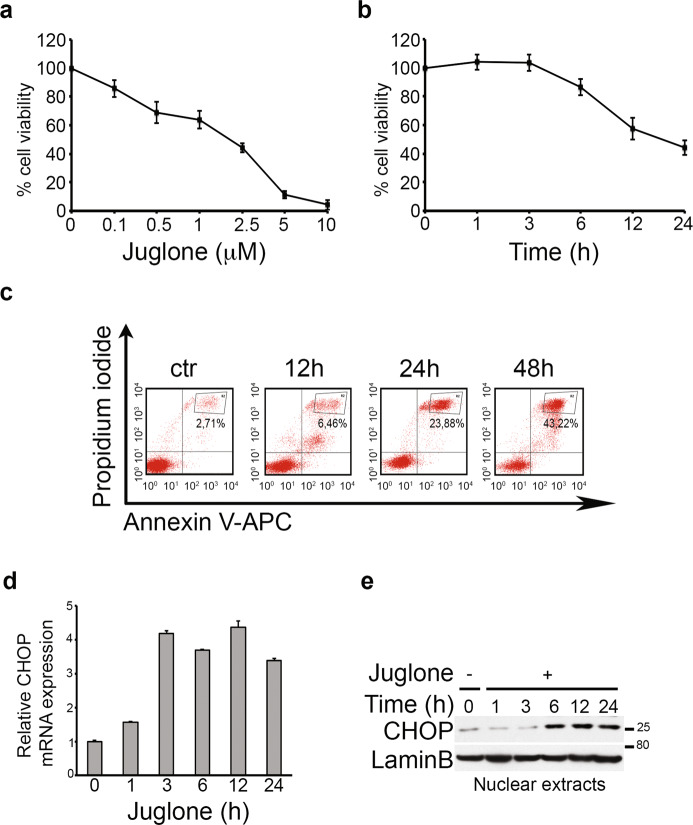
*Juglone* induces cytotoxic effects in Notch3-overexpressing TALL-1 cells via apoptosis, ER-associated. **a** Cell count of TALL-1 cells treated with increasing doses of *Juglone* for 24 h (IC_50_: 1.8). **b** Cell count of TALL-1 cells treated with a fixed-dose (2.5 μM) of *Juglone* for the times indicated (h). **c** Flow cytometric analysis of Annexin V-APC/PI-stained TALL-1 cells treated with 2.5 μM of *Juglone* for the times indicated (h). The percentages of late apoptotic cells (Annexin V-APC+/PI+, top right quadrant, gate R2) are indicated. ctr, untreated cells. **d**, **e** Relative mRNA expression (**d**) and nuclear protein expression (**e**) of CHOP after 2.5 μM of *Juglone* treatment of TALL-1 cells for the time indicated (h). Anti-Lamin B was used as a nuclear fraction marker. All data are representative of at least three independent experiments, each in triplicate.

Several in vitro experiments were first performed to test the multiple effects of *Juglone* on TALL-1 cells. Since it has been shown that *Juglone* induces apoptosis in several cancer cells^37,38^, including leukemia^11^, we first investigated its effects on TALL-1 leukemic cells survival: as shown in the Fig. 4a, increasing doses of *Juglone* for 24 h correlated with progressive cell growth inhibition. The time-dependent anti-proliferative effects of a fixed dose of *Juglone* are shown in Fig. 4b. An increase in the percentage of late apoptotic cells (positive for both Annexin V and PI) after 12 h, progressively increasing up to 48 h was observed upon *Juglone* treatment (Fig. 4c). Notably, regarding ER stress/UPR balance, we observed the increased expression of CHOP, both at mRNA (Fig. 4d) and nuclear protein levels (Fig. 4e), thus suggesting the involvement of ER stress in the *Juglone*-induced leukemia cell death. Similar results were obtained from Juglone-treated N3-232T murine T lymphoma cells, we previously established from N3ICtg mice, which represents the murine counterpart of TALL-1 cells as they display the constitutive activation of Notch3 in the absence of Notch1-activating mutations and activation^39^ (Supplementary Fig. S6a–d).

Several cellular stimuli that perturb ER homeostasis may lead to ER stress, including proteasome function alteration^40^, increase of reactive oxygen species (ROS)^41^, and [Ca^+2^]_ER_ depletion^42^. In TALL-1 cells, we observed that *Juglone* treatment was able to induce at the same time: (1) the fast accumulation of poly-ubiquitinated proteins (Supplementary Fig. S7a); (2) the increase in the Ca^+2^ release from the ER to the cytoplasm (Supplementary Fig. S7b), probably due to the significant inhibition of the mRNA expression of sarco(endo)plasmic reticulum Ca^+2^ ATPase 3 (SERCA3), known to pump cytoplasmic Ca^+2^ to ER lumen and to be expressed at high levels in the hematopoietic cell lineage^43^ (Supplementary Fig. S7c). All these observations sustained the ability of *Juglone* to induce ER stress through different mechanisms: the increased GRP78/Bip expression, both at mRNA (Supplementary Fig. S7d) and protein (Supplementary Fig. S7e) levels, also observed in combination with the widely used ER stress inducer Thapsigargin, TH (Juglone + TH) (Supplementary Fig. S7e), validated these data. As a consequence, flow cytometric analysis of Annexin V/PI-staining showed that the drug combination (Jug + TH) was more effective than each single agent in inducing apoptosis, as demonstrated by the increase in the percentage of late apoptotic cells (Supplementary Fig. S7f) and confirmed by the significant decrease in cell count (Supplementary Fig. S7g).

*Juglone* is a natural inhibitor of Pin1 protein, a peptidyl-prolyl isomerase that we recently discovered as a novel regulator of Notch3-IC protein expression in T-ALL^36^. Furthermore, we and others demonstrated its ability to inhibit SERCA activity (Supplementary Fig. S7c and ref. ^35^). Since SERCA inhibition can modulate Notch function in T-ALL cell lines by affecting Notch1 maturation process^44^, we supposed the potential co-presence of Pin1-independent mechanisms mediating *Juglone* function upon Notch proteins inhibition in T-ALL, as previously described in different cellular contexts^45^. As expected, reduced levels of Notch3 receptor at the cell surface (N3EC) (Supplementary Fig. S7h), resulting in the reduced Notch3 protein expression (Supplementary Fig. S7i), were observed in *Juglone*-treated TALL-1 cells with respect to the control cells (Supplementary Fig. S7h–i).

Together, these findings supported the possibility of using *Juglone* in order to induce TALL-1 cells death through the simultaneous Notch3 downregulation and ER stress induction.

### *Juglone* affects ER stress/UPR signaling by regulating IRE1α protein expression

**Fig. 5:**
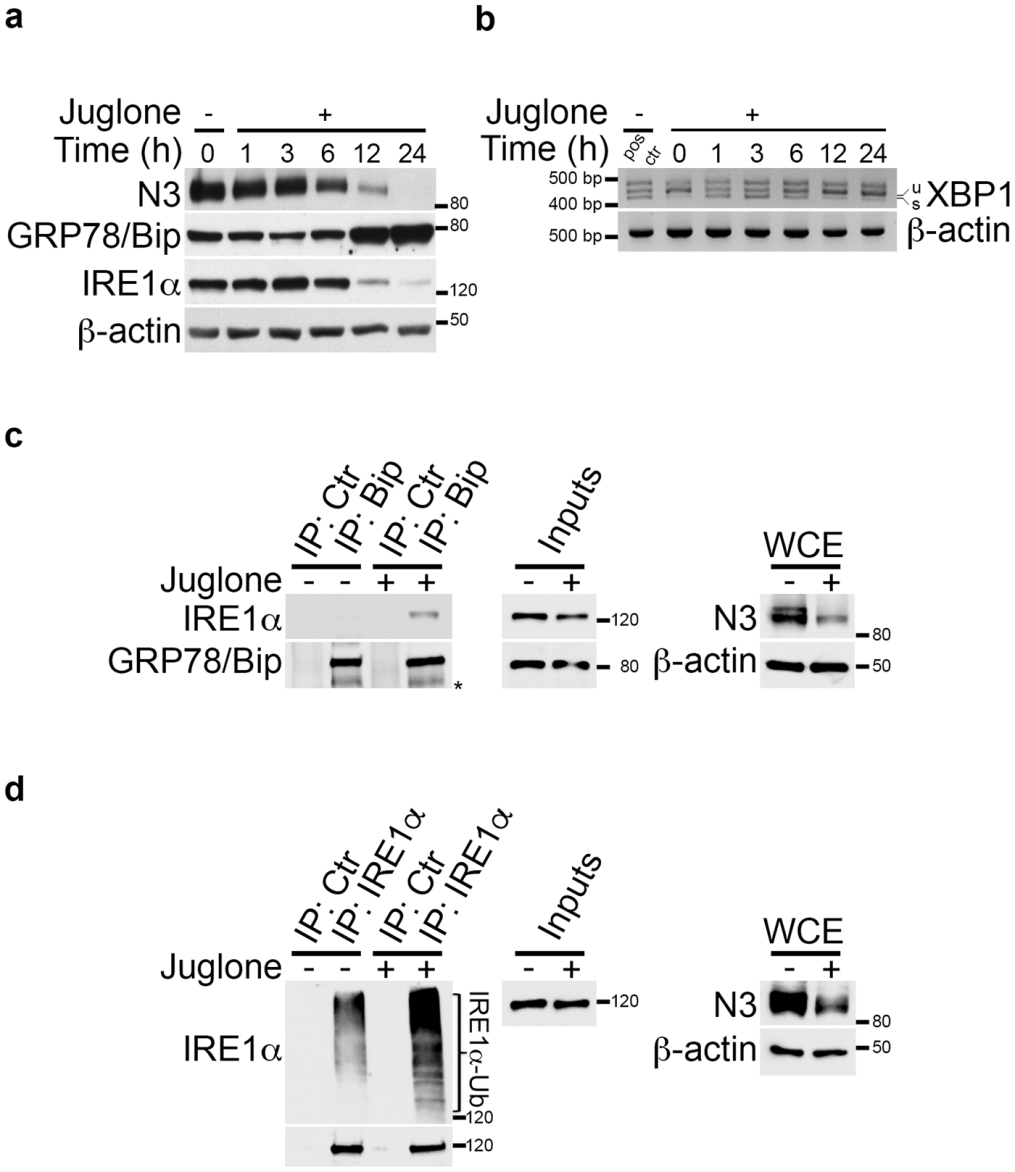
*Juglone* treatment modulates ER stress/UPR signaling by recapitulating the combined Notch3 downregulation under ER stress conditions. **a** Western blot analysis showing the time-dependent modulation of Notch3 (N3), GRP78/Bip and IRE1α protein expression after *Juglone* treatment (2.5 μM) of TALL-1 cells. **b** RT-PCR analysis of XBP1 mRNA derived from TALL-1 cells described in **a**. u unspliced XBP1; s spliced XBP1; pos ctr positive control, cells treated with Thapsigargin for 24 h. **c** Control or anti-GRP78/Bip (Bip) immunoprecipitates from TALL-1 cells treated or not with 2.5 μM *Juglone* for 6–8 h were subjected to western blot and probed with anti-IRE1α and anti-GRP78/Bip antibodies to detect endogenous GRP78/Bip-IRE1α and GRP78/Bip immunoprecipitated protein levels, respectively. *non specific band?. **d** Control or anti-IRE1α immunoprecipitates from the same cells used in **c** were probed with an anti-Ubiquitin (Ub) and anti-IRE1α antibodies to detect the Ubiquitination status of IRE1α and IRE1α immunoprecipitated protein levels, respectively. In both **c** and **d**, proteasomal inhibition with MG132 was used. The input lanes of the panels **c** and **d** show 5% of total lysate. Whole-cell extracts (WCE) analysis was used to control Notch3 (N3) downregulation after *Juglone* treatment. In all panels, **a**, **c**, and **d** the anti-β-actin was used as a loading control. All data are representative of at least three independent experiments, each in triplicate.

Since we have demonstrated that Notch3 downmodulation under ER stress conditions may be correlated with a UPR defect (Fig. 3), we further evaluated the *Juglone* effects upon the ER stress/UPR signaling. As shown in the Fig. 5a, the levels of ER stress/UPR markers were not significantly altered until 6 h of *Juglone* treatment, while they were strongly modulated after 12–24 h: in particular, *Juglone* significantly decreased the expression of Notch3 and IRE1α proteins while increasing GRP78/Bip levels (Fig. 5a and Supplementary Fig. S6e). In keeping with these results, similar time-dependent kinetics were observed for the ER stress-activated XBP1 splicing, as demonstrated by the modulation of the XBP1 spliced band (XBP1s) during *Juglone* treatment (Fig. 5b): at very early time points (1–3 h) XBP1s was produced while disappeared progressively at 12–24 h (Fig. 5b), possibly due to the observed defect in IRE1α expression (Fig. 5a).

All these data raised the possibility that *Juglone* might also be able to suppress UPR at later time points through the regulation of the Notch3-GRP78/Bip-IRE1α axis previously described (Fig. 3). As expected, *Juglone* treatment, by downregulating Notch3 and overexpressing GRP78/Bip at the same time, was able to restore the GRP78/Bip-IRE1α interaction (Fig. 5c), which in turn correlated with increased IRE1α ubiquitylation (Fig. 5d) and inactivation, thus confirming previous mechanistic data (Fig. 3) and finally pointing out the potential involvement of Notch3 in the *Juglone*-dependent ER stress/UPR signaling regulation.

Notably, interesting results obtained from patient-derived T-ALL cells (PDTALLs)^17^ (Supplementary Fig. S8a) suggested the potential clinical relevance of the *Juglone*, as we observed that *Juglone* treatment is able to reduce Notch3 protein expression and to induce T-ALL cell death in a dose-dependent manner (Supplementary Fig. S8b). Interestingly, in PDTALLs expressing Notch3 (PDTALL6 and 8), but not in the absence of Notch3 (PDTALL13), *Juglone* significantly decreased the expression of IRE1α protein while increasing GRP78/Bip levels (Supplementary Fig. S8c), thus confirming previous in vitro studies.

### Notch3 silencing amplifies the ER stress-associated pro-apoptotic effects of the *Juglone* in TALL-1 cells

**Fig. 6:**
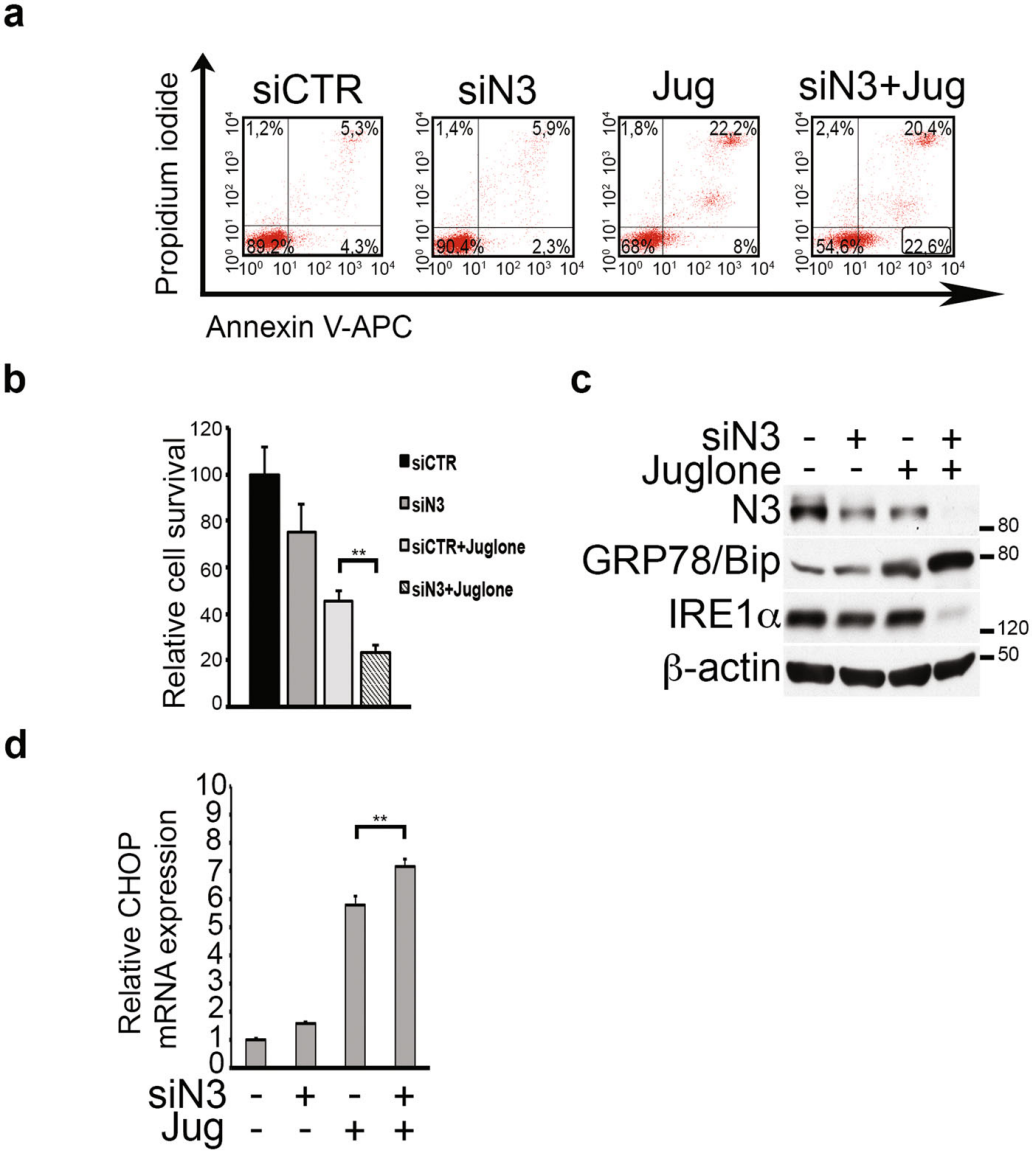
Notch3 silencing contributes to amplify *Juglone*-dependent ER stress-associated apoptosis. **a** Flow cytometric analysis of Annexin V-APC/PI-stained TALL-1 cells, Notch3-silenced for 72 h and treated for the last 24 h with *Juglone* showed that the combined treatment was more effective in inducing apoptosis than single treatments. The percentages of early apoptotic cells (Annexin V-APC+/PI−, bottom right quadrant) and late apoptotic cells (Annexin V-APC+/PI+, top right quadrant) are indicated. siCTR cells treated with scramble siRNA; siN3 Notch3-silenced cells; Jug *Juglone*; siN3+Jug combined samples. Data shown are representative of three independent experiments performed in triplicate. **b** Relative cell survival of TALL-1 cells derived from the experiments described in **a** with Notch3 silencing prolonged for 96 h. The combined treatments resulted in a discrete synergism (EOB: 13 ± 2). EOB value was calculated as the mean ± SD of at least three independent experiments. **c** Western blot analysis of Notch3-silenced TALL-1 cells treated for the last 24 h with *Juglone* showed that the Notch3 silencing synergized both in increasing GRP78/Bip expression and in decreasing IRE1α expression. Anti-β-actin was used as a loading control. **d** Relative CHOP mRNA expression derived from TALL-1 cells described in **c**. For **b** and **d** results are shown as the means average deviations of at least three separate experiments and *P*-values were calculated using Student’s *t*-test (i.e., ns not significant; **P ≤ 0.01).

We have demonstrated that the mechanism of *Juglone*-induced leukemia cell death could involve an induction/amplification of an ER stress microenvironment, followed by a serious defect of the ER stress response, as evidenced by the strong downregulation of IRE1α expression and function observed at late time points (Fig. 5). This scenario may render leukemic cells unable to adequately respond to ER stress, thus finally leading to the cell-destroying pathway activation which prevails over the compensatory UPR (Fig. 4). To define how Notch3 contributes to *Juglone*-induced T-ALL cell apoptosis, we examined the existence of a potential synergism between *Juglone* and the decrease of Notch3, by using Notch3-silenced TALL-1 cells. The absence of Notch3 in *Juglone*-treated (siN3 + Jug) cells (thus under ER stress conditions) resulted in an increased percentage of early apoptotic cells (Fig. 6a), when compared to the cells treated with *Juglone* alone (Jug), which in turn promoted a significant decrease in cell count when Notch3 silencing was further prolonged for 96 h (Fig. 6b). More interestingly, the silencing of Notch3 strongly synergized with *Juglone* treatment in both increasing GRP78/Bip expression and decreasing IRE1α expression (Fig. 6c and Supplementary Fig. S9a). These data reinforce the potential role of Notch3 inhibition in the *Juglone*-dependent perturbation of ER stress/UPR signaling, which finally leads to a stronger ER stress-associated cell apoptosis induction, as demonstrated by the significant concomitant amplification of the mRNA levels of CHOP (Fig. 6d and Supplementary Fig. S9b).

Notably, despite the expected Notch1 downregulation observed after *Juglone* treatment, related to its previously described SERCA inhibition function (Supplementary Fig. S7c and ref. ^44^), Notch1 silencing in the selected Notch3^low/neg^ cells (Jurkat and Ke37) did not amplify the decreased cell count induced by *Juglone* itself (Supplementary Fig. S10a, b, left panels). The possible reason for the different behavior of Notch1 with respect to Notch3 could be related to the inability of Notch1 to interact with GRP78/Bip protein (Supplementary Fig. S10c), independently of its mutational and activational *status*. In keeping with these observations, no significant change of IRE1α expression was observed in Notch1-silenced T-ALLs analyzed after *Juglone* treatment (Supplementary Fig. S10a,b, right panels) and no significant correlation was found from the *in silico* analysis of the Notch1 and IRE1α gene expression levels in the same cohorts of T-ALL-bearing patients shown in Fig. 3e (Supplementary Fig. S10d). Therefore, these findings excluded the involvement of Notch1 in the mechanistic hypothesis of GRP78/Bip sequestration, which seems to be specifically related to a novel and unknown role of Notch3, finally aimed to sustain the UPR signaling in the T-ALL context.

In support of this exclusive ability, we observed an higher cell survival decrease of *Juglone*-treated T-ALLs expressing Notch3 (TALL-1, Molt3, DND41, SIL-ALL, P12-I) when compared to Notch3 negative/low cells (Jurkat, KOPKT1, Ke37) (Supplementary Fig. S11), thus suggesting that Notch3 expression levels may dictate the T-ALLs sensitivity to *Juglone*.

### Juglone displays in vivo activity against TALL-1 tumor growth models by defecting Notch3 expression and inducing ER stress-associated apoptosis

**Fig. 7:**
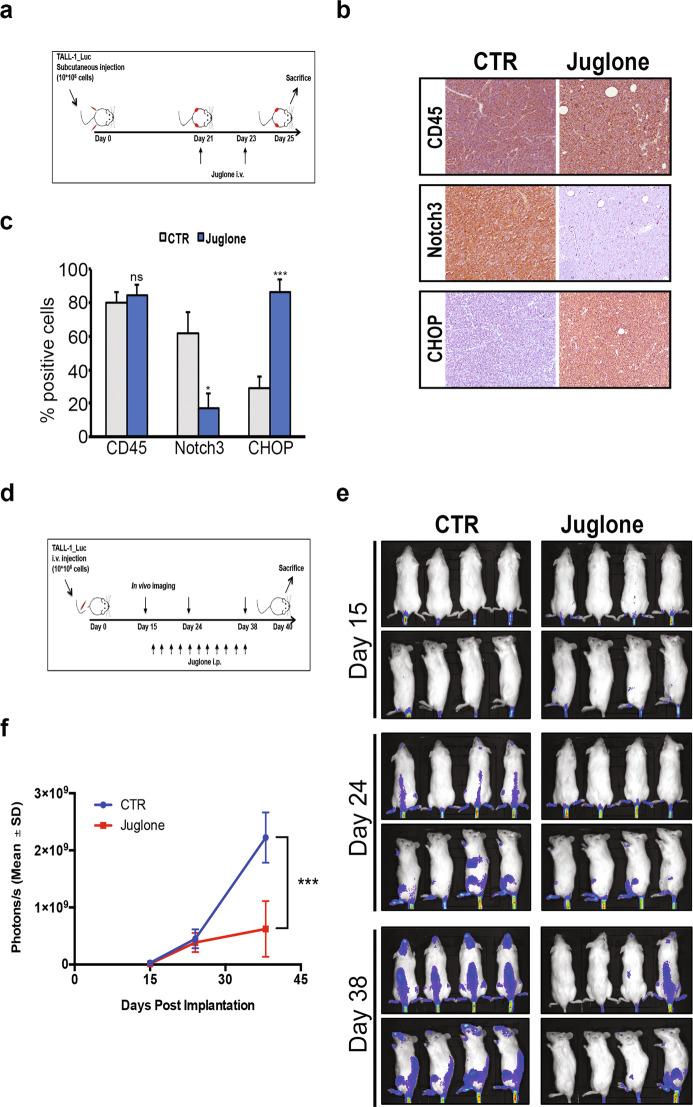
*Juglone* reduces tumor burden in mice-bearing TALL-1_luc cells. **a** Outline of treatment with Juglone in NSG mice-bearing TALL1_luc cells subcutaneously injected at day 0. Mice received two intravenous injections (i.v.) of *Juglone* (1 mg/kg) or vehicle (CTR) at days 21st and 23rd. Mice were killed at day 25th (*n* = 6 each group). **b** Tumors were then harvested, fixed in formalin and analyzed by immunohistochemical staining with the antibodies for CD45 (upper panels), Notch3 (middle panels), and CHOP (lower panels). **c** Percentages of positive cells for the indicated stainings described in **b**. Results are shown as the means average deviations of three separate experiments and *P*-values were calculated using Student’s *t*-test (i.e., ns not significant *P* > 0,05; *P ≤ 0.05; ***P ≤ 0.001). **d** Outline of treatment with Juglone in NSG mice-bearing TALL1_luc cells intravenously injected (i.v.) at day 0. Mice received intraperitoneal injections (i.p.) of *Juglone* (1 mg/kg) or vehicle (CTR) every 2 days, starting from day 16th until day 38th. Mice were killed at day 40 (*n* = 6 each group). **e**, **f** Tumor size were monitored with the Xenogen in vivo imaging system, as reported in the “Material and methods” section. Representative images (**e**) and quantitative analysis (**f**) of luciferase activity in mice treated with Juglone or vehicle alone (CTR) at days 15th, 24th, and 38th from TALL-1_luc injection are shown. Statistically significant differences in average radiance in the two groups of samples are indicated on day 38th. *P*-values were calculated using Student’s *t*-test (i.e., ***P ≤ 0.001).

The effects of the *Juglone* activity upon Notch3 protein expression were also confirmed in a human T-ALL xenograft mouse model. Mice were treated with intravenous injection of *Juglone* or vehicle alone (CTR) at days 21th and 23th after subcutaneous leukemia cells implantation (Fig. 7a) and, on day 25th, excised tumors were evaluated for the effects on Notch3 and CHOP expression (Fig. 7b,c). As expected, comparable levels of CD45, used as a marker of human TALL-1 injected cells, were observed between tumors after the short treatment performed (Fig. 7b, upper panels and Fig. 7c). Notably, staining of xenografts with anti-Notch3 antibody revealed a strong reduction of Notch3 expression following *Juglone* treatment with respect to the high levels observed in xenografts from animals treated with vehicle alone (Fig. 7b, middle panels, and Fig. 7c). Interestingly, significantly increased CHOP levels were observed in *Juglone*-treated tumors when compared to controls (Fig. 7b, lower panels, and Fig. 7c). These studies demonstrated that *Juglone* treatment is able to inhibit Notch3 expression in vivo, thus recapitulating the previously described perturbation of the ER stress/UPR signaling balance.

Based on the observed strong in vitro activity of *Juglone* on TALL-1 cell survival, we wondered whether it also has antitumor effects in a TALL-1 xenograft mouse model, by using TALL-1 cells luciferized (TALL-1_luc) in order to evaluate xenograft growth in vivo.

Mice were injected intravenously (i.v.) with TALL-1_luc cells and were monitored by optical imaging at various time points after cells implantation (day 0): on day 15th after cell transfer, mice were randomly distributed in two groups to receive one intraperitoneal (i.p.) injection of *Juglone* or vehicle alone (CTR) every two days, starting from day 16th until day 38th, and were sacrificed at day 40th (Fig. 7d). Results showed a strong and significant reduction in leukemia burden in *Juglone*-treated mice with respect to control mice at all time points analyzed (Figs. 7e, f).

Taken together, our data suggest that the *Juglone*-dependent inhibition of Notch3 might be a useful therapeutic strategy for Notch3-overexpressing T-ALLs.

## Discussion

Increasing evidence supports an important role of the UPR in cancer through the identification and characterization of mechanisms by which tumor cells are able to promote their own survival in unfavorable conditions, leading to tumor progression and metastasis^46^. In this view, targeting the UPR represents a new window of research focused on the identification of druggable targets against malignancies, including acute leukemia^7^. The therapeutic potential of targeting UPR signaling in cancer could involve two main approaches: 1. inhibition of UPR to eradicate tumors that are strongly dependent on an activated UPR for their survival in unfavorable conditions (i.e., highly stressed ER) or 2. induction of a severe ER stress by the accumulation of misfolded protein in the ER in order to overload restoration ability of tumor cells with compromised UPR or to hyper activate the UPR to kill cells through pro-apoptotic UPR signaling^46^.

Small molecule inhibitors that target the UPR transducers (i.e., PERK/eIF2α and IRE1α/XBP1 signaling axis) are currently available^47–49^ and several compounds are found to block the functional activity of GRP78/Bip protein^50,51^, whose high levels are commonly related to tumor protection, survival and chemoresistance^52^. In addition, it has been well documented that proteasome inhibitors may potentiate ER stress in cancer cells, thus promoting proteotoxic conditions^52^. Combined therapies are also under investigation: the proteasome inhibitor Bortezomib combined with small molecules that inhibit IRE1α activity significantly decreased Multiple Myeloma growth in vivo^53^ as well as combining Bortezomib with the SERCA inhibitor Thapsigargin amplified ER stress and increased cancer cell death^54^. In the T-ALL context, it has been demonstrated that pharmacological inhibition of Casein Kinase 2 (CK2) through CX-4945 may be an efficient treatment for a subset of T-ALLs displaying upregulation of the CK2/PI3K/Akt/mTOR axis via downmodulation of the ER stress/UPR signaling^55^. More recently, Huiting and colleagues clearly documented the cross-talk between MYC and UFD1, a component of the ER-associated degradation complex (ERAD) commonly involved in the pro-survival UPR signaling^56^.

In this study, we demonstrated that Notch3 may play a novel role in T-ALL, being important in sustaining the UPR through the regulation of IRE1α protein expression and function. In addition, since it has been recently demonstrated that IRE1α is able to up-regulate its own transcription through a positive regulation loop^57^, evaluating Notch3 and IRE1α correlation at mRNA levels *per se* may represent an important feature of T-ALL-bearing patients that rely on UPR through the Notch3-IRE1α axis activation to survive, thus finally predicting a potential novel therapeutic target. The significant positive correlation observed between Notch3 and IRE1α mRNA expression levels in human T-ALL primary tumor samples confirms the possible relevance of our observations for human T-ALL development. Previous data sustained the role of proteasome inhibitors (i.e., Bortezomib) in disrupting the ER stress response in Myeloma cells by suppressing the endoribonuclease function of IRE1α, through an unknown mechanism possibly involving unknown protein(s) that may act by stabilizing IRE1α-GRP78/Bip association^58^. In keeping with these findings, in this work, we observed that Notch3 (but not Notch1) is able to interact with GRP78/Bip in leukemia cells in basal conditions. As a consequence, the absence of Notch3 induced under ER stress conditions lets GRP78/Bip (overexpressed in the ER stress microenvironment) free to interact with IRE1α, thus leading to its ubiquitination and inactivation, as recently described^31^. Interestingly, an *in silico* analysis we performed on T-ALL cell lines identified a significant correlation between Bortezomib drug sensitivity and Notch3 expression levels (Sanger GDSC2:1191 assay) (data not shown), thus indicating a novel relationship, which supports our data but it needs to be further investigated.

All these data suggested the potential use of Notch3 targeting in combination with an ER stress inducer as a novel therapeutic approach of a subset of Notch3-overexpressing T-ALLs that rely on UPR for their survival. Such an approach would be based on the disruption of the pro-survival UPR signaling, partially represented by the Notch3-dependent IRE1α/XBP1 axis, thus forcing to switch to UPR pro-apoptotic mode, mainly promoted by the detectable increase of pro-apoptotic CHOP expression. To this purpose we exploited the observed abilities of the natural compound *Juglone* to act simultaneously as 1. an ER stress aggravator (ERSA), thus exacerbating ER stress conditions in TALL-1 cells and 2. a Notch3 inhibitor, thus defecting UPR by IRE1α downmodulation.

Finally, all these events contribute to the *Juglone*-dependent leukemia cell death via CHOP induction, also observed in vivo, thus confirming previous data obtained after treatment of tumor-bearing mice with ERSA compounds^54^.

**Fig. 8:**
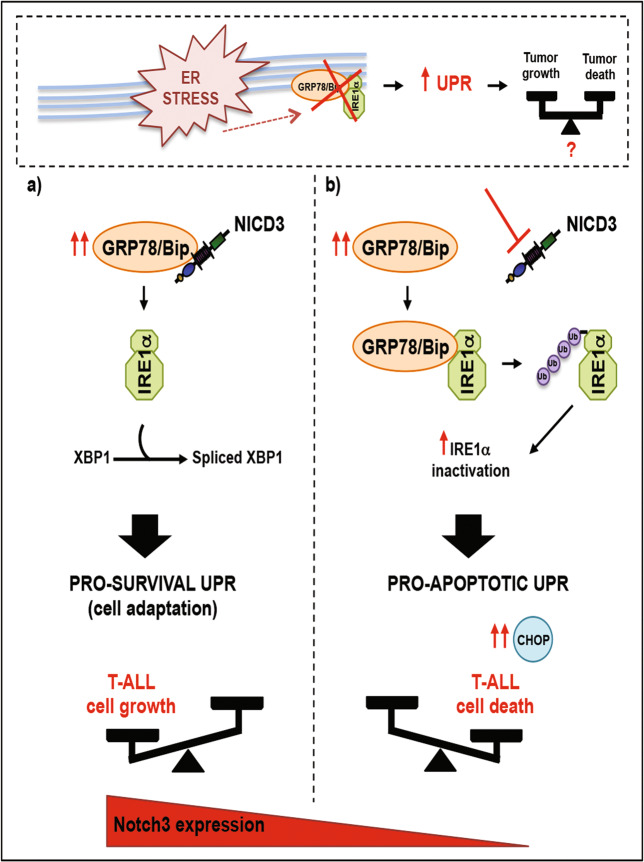
Schematic diagram to summarize our working model. Under ER stress conditions, IRE1α can be activated through the release of the known inhibitory GRP78/Bip-IRE1α binding, thus leading to the UPR activation (i.e., increased expression of the master gene GRP78/Bip), which can result in tumor survival or tumor death. **a** A tumor cell overexpressing Notch3 under ER stress conditions: Notch3 interacts with GRP78/Bip thereby sustaining IRE1α constitutive activation which contributes to favor a pro-survival UPR, finally resulting in maintaining T-ALL cell growth. **b** A tumor cell treated with Notch3 blocking agents under ER stress conditions (i.e., *Juglone*): the absence of Notch3 lets the overexpressed GRP78/Bip free to interact with IRE1α, thereby promoting IRE1α ubiquitination and inactivation, thus contributing to the switch to a pro-apoptotic UPR through increasing CHOP levels and finally resulting in T-ALL cell death. NICD3 Notch3 intracellular domain.

*Juglone* treatment provided new insights unveiling a possible development of more effective therapies, exploiting the idea of aggravating ER stress and defecting UPR at the same time, thus preventing leukemic cells from engaging a functional UPR to restore ER homeostasis through Notch3 protein modulation. To our knowledge, this is the first study demonstrating a specific involvement of Notch3 in regulating the balance between UPR pro-survival and UPR pro-death under ER stress conditions. These findings suggest that, in addition to the currently established approaches^5^, the modulation of the ER stress/UPR signaling through a selective Notch3 inhibition could be exploited for inducing T-ALL cell death (Fig. 8), thus improving the outcome of Notch3-dependent TALL-bearing patients. Our in vivo studies performed with chronic administration of *Juglone* showed significant inhibition of Notch3-dependent leukemia growth through Notch3 downregulation, thus providing preclinical evidence of the efficacy of Notch3 targeting in T-ALL, which is becoming of great interest as a potential novel therapeutic approach^15,59,60^. Like other ERSAs compounds (i.e., thapsigargin, tunicamycin, brefeldin A,…) which are under investigation for anticancer applications in clinics^52^, *Juglone* may represent a new agent whose cancer therapeutic efficacy should be considered. In this regard, data obtained with PDTALL cells, even if they require further validation studies due to the limited sample size currently available, provide evidence that *Juglone* treatment may be also relevant in clinical applications. In order to include *Juglone* in the natural compounds modulating Notch pathway^61^, further studies will be required to fully understand its mechanism of action and to enable selective and target-specific drug delivery, in keeping with what it has been shown for similar compounds such as Thapsigargin^62^, thus reducing drug-related toxicities. In this scenario, *Juglone* efficacy could be further increased via combination with Notch3 inhibitors, as we showed that Notch3 silencing in TALL-1 cells amplifies the ER stress-associated pro-apoptotic effects of the *Juglone*, thus resulting in antitumor synergy effects with potential lower toxicity. Moreover, since we demonstrated the importance of Notch3 in T-ALL progression^36^, confirming the same role observed also in solid tumors^63,64^, we can speculate that *Juglone*-dependent Notch3 inhibition could be useful also for tumors that do not depend on Notch3 at their onset but that could recur and become more aggressive subsequently, due to a selective growth advantage represented by the Notch3-dependent UPR maintenance. In this view, further studies are required to identify Notch3-overexpressing tumors where the aggravation of ER stress plus Notch3 depletion, *Juglone*-induced, could be particularly beneficial.

## Supplementary information

Supplementary Information

Supplementary Figures and Table Legends

Supplementary Materials and Methods

Supplementary Figure S1

Supplementary Figure S2

Supplementary Figure S3

Supplementary Figure S4

Supplementary Figure S5

Supplementary Figure S6

Supplementary Figure S7

Supplementary Figure S8

Supplementary Figure S9

Supplementary Figure S10

Supplementary Figure S11

Supplementary Table S1
