# Supplementary material for: Notch3 contributes to T-cell leukemia growth via regulation of the unfolded protein response
Source: Oncogenesis. 2020 Oct 18;9(10):93. doi: 10.1038/s41389-020-00279-7 (PMC7569087; doi:10.1038/s41389-020-00279-7)
Supplement: Supplementary file 1 — Supplementary Information [file 41389_2020_279_MOESM1_ESM.docx]

In the Supplementary Informations there are included 14 files, as follow:

- Supplementary Figure S1 (.tif)

- Supplementary Figure S2 (.tif)

- Supplementary Figure S3 (.tif)

- Supplementary Figure S4 (.tif)

- Supplementary Figure S5 (.tif)

- Supplementary Figure S6 (.tif)

- Supplementary Figure S7 (.tif)

- Supplementary Figure S8 (.tif)

- Supplementary Figure S9 (.tif)

- Supplementary Figure S10 (.tif)

- Supplementary Figure S11 (.tif)

- Supplementary Table S1 (.xlsx)

- Supplementary Figures and Table Legends (.docx)

- Supplementary Materials and Methods (.docx)
