# Supplementary material for: Notch3 contributes to T-cell leukemia growth via regulation of the unfolded protein response
Source: Oncogenesis. 2020 Oct 18;9(10):93. doi: 10.1038/s41389-020-00279-7 (PMC7569087; doi:10.1038/s41389-020-00279-7)
Supplement: Supplementary file 2 — Supplementary Figures and Table Legends [file 41389_2020_279_MOESM2_ESM.docx]

**Supplementary Figure S1. Differential Notch1 and Notch3 expression in T-ALL cell lines**

a) Western blot analysis of Notch3 (N3) and Notch1 (N1) in T-ALL cells (TALL-1; Ke37; KOPKT1; p12-Ichikawa; SIL-ALL; Jurkat; DND41; Molt3). β-actin was used as loading control.

**Supplementary Figure S2. Notch expression in T-ALL cell lines after Tunicamycin treatment**

a-f) Representative Western blots of Notch3 (N3) and Notch1 (N1) in TALL-1 (a,b), Molt3 (c,d) and DND41 (e,f) cells after 20μM Tunicamycin treatment shown in the Figures 2a-f, to control the quality of Notch silencing. β-actin was used as loading control. #1: first sequence of siRNA; #2: second sequence of siRNA.

**Supplementary Figure S3. Notch3 modulation in Tunicamycin-treated TALL-1 cells does not affect ATF6 cleavage and PERK expression**

a) Left panel, Western blot analysis of Notch3-silenced (siN3) TALL-1 cells treated with Tunicamycin (Tun) for 24 hours showed that drugs unaffected ATF6 cleavage and PERK expression. Right panels, Optical Densitometry (OD) of PERK and c-ATF6 protein expression levels analyzed in all the experiments performed (at least 3 biological replicates), thus including the p-values, calculated using Student’s T-test (i.e., ns, not significant P>0,05). c: cleaved ATF6; t: total ATF6. The OD values for c-ATF6 included in the graph were calculated as ratio c-ATF6/t-ATF6, after setting arbitrarily the OD values of the missing bands to 1. The number of the lanes correspond to: 1: siCTR; 2: siN3; 3: Tun; 4. siN3+Tun.

**Supplementary Figure S4. Notch3 silencing alone does not influence the ER stress/UPR markers expression**

 a-c) With respect to control cells, Notch3-silenced TALL-1 cells (siN3) showed similar mRNA expression levels of GRP78/Bip (a), similar protein expression of GRP78/Bip and IRE1α (b) and similar splicing of XBP1 mRNA (c): u, unspliced XBP1; s, spliced XBP1; pos ctr: positive control, cells treated with Thapsigargin for 24 hours (left panel). c, right panel: Optical Densitometry (OD) of spliced/unspliced ratio of XBP1 levels analyzed in all the experiments performed (at least 3 biological replicates), including the p-values, calculated using Student’s T-test (i.e., ns, not significant ). For panel (a) results are shown as the means average deviations of at least three separate experiments and p-values were calculated using Student’s T-test (i.e., ns, not significant).

**Supplementary Figure S5. Notch3 co-immunoprecipitates also in Notch3 wild-type (wt) T-ALL expressing cells**

a) Control or anti-GRP78/Bip (Bip) immunoprecipates from Jurkat cells were subjected to western blot and probed with anti-N3 antibody to analyse the endogenous GRP78/Bip-Notch3 interaction. The blot with anti-GRP78/Bip antibody was used to detect the GRP78/Bip immunoprecipitated protein levels. The input lanes show 5% of total lysate.  Data are representative of at least three independent experiments, each in triplicate.

**Supplementary Figure S6. Juglone induces cytotoxic effects in Notch3-overexpressing murine N3-232T cells via apoptosis, ER-associated**

a) Cell count of N3-232T cells treated with increasing doses of *Juglone* for 24 hours (h) (IC_50_: 1,4). b) Cell count of N3-232T cells treated with a fixed dose (2.5μM) of *Juglone* for the times indicated. c) Flow cytometric analysis of Annexin V-APC/PI-stained N3-232T cells treated with a fixed dose (2.5μM) of *Juglone* for the times indicated (h). The percentages of late apoptotic/necrotic cells (Annexin V-APC+/PI+, top right quadrant, gate R2) are indicated. ctr, untreated cells. d) Nuclear protein expression of CHOP after *Juglone* treatment of N3-232T cells for the time indicated. Anti-Lamin B was used as a nuclear fraction marker. e) Western blot analysis showing the time-dependent modulation of Notch3 (N3), GRP78/Bip and IRE1α protein expression after *Juglone* treatment (2.5μM μM) of N3-232T cells. Anti–β-actin was used as a loading control.

All data are representative of at least three independent experiments, each in triplicate.

**Supplementary Figure S7. *Juglone* simultaneously induces ER stress and downregulates Notch3 through different mechanisms**

a) Time course analysis of *Juglone* treatment (2.5μM) on TALL-1 cells showing the fast increase of poly-ubiquitinated proteins. b-c) *Juglone* treatment (2.5μM) of TALL-1 cells and analysis of b) intracellular calcium concentration, [Ca+2 ] and c) mRNA levels of SERCA3, as described in the Material and Methods section.  d) Relative mRNA expression of GRP78/Bip after *Juglone* treatment of TALL-1 cells for the time indicated (h). e) Western blot analysis of TALL-1 cells treated for 24 hours with *Juglone* and Thapsigargin (TH), either alone or in combination (Jug+TH), showed that the combined treatment synergized in increasing GRP78/Bip. Anti–β-actin was used as a loading control. *non specific band?.  f) Flow cytometric analysis of Annexin V-APC/PI-stained TALL-1 cells, treated for 24 hours as in (e) showed that the combined treatment was more effective in inducing apoptosis than single treatments. The percentages of early apoptotic cells (Annexin V-APC+/PI-, bottom right quadrant) and late apoptotic/necrotic cells (Annexin V-APC+/PI+, top right quadrant) are indicated. Data shown are representative of three independent experiments performed in triplicate. g) Relative cell survival of TALL-1 cells derived from the experiments described in e-f). h) Cytofluorimetric analysis of Notch3 extracellular expression (N3EC) from TALL-1 cells after treatment with *Juglone*, indicated as percentages inside each quadrant. The black curve represent the isotypic control. Ctr, untreated cells. i) Western blot analysis of extracts from the same TALL-1 cells described in h) and probed with anti-Notch3-EC (N3EC) and anti-Notch3 (N3) antibodies to evaluate the Notch3 protein expression after drug treatment. The β-actin expression was used as loading control. FL: Notch3 full-lenght (280kDa); EC: Notch3 extracellular (220kDa).  All data are representative of at least three independent experiments, each in triplicate. In both panels c) and g) results are shown as the means average deviations and p-values were calculated using Student’s T-test (i.e. *P≤0.05; **P≤0.01; ***P≤0.001).

**Supplementary Figure S8. Patient-derived TALL analysis**

a) Screening of Notch3 protein expression (N3) in primary TALL patients (PDTALL). b) PDTALL 6 analysis: (left panel) Flow cytometric analysis of Annexin V-APC/PI-stained cells treated with increasing doses of *Juglone* for 24 hours; ctr, untreated cells. (right panel) Western blot analysis to evaluate Notch3 expression after 2.5μM of *Juglone*. c) Western blot analysis of PDTALL Notch3-positive (PDTALL 6 and 8) and Notch3-negative (PDTALL 13) cells after treatment with 2.5μM of *Juglone* in order to evaluate the changes in UPR *status* (IRE1α and GRP78/Bip expression levels); ctrl, extract from TALL-1 cells.

In all the western blots included in the Figure the anti–β-actin was used as a loading control.

**Supplementary Figure S9. Notch3 silencing contributes to amplify *Juglone*-dependent ER-stress associated apoptosis**

a) Western blot analysis of Notch3-silenced TALL-1 cells, obtained by using a second siRNA sequence (siN3 #2) and treated for the last 24 hours with 2.5μM of *Juglone,* confirmed that Notch3 silencing synergized both in increasing GRP78/Bip expression and in decreasing IRE1α expression. Anti–β-actin was used as a loading control. b) Relative CHOP mRNA expression derived from TALL-1 cells described in (a). Results are shown as the means average deviations of three separate experiments and p-values were calculated using Student’s T-test (i.e., **P≤0.01).

**Supplementary Figure S10. Modulation of Notch1 expression in T-ALLs does not influence the *Juglone*-dependent ER-stress associated apoptosis**

a-b) Notch1-silenced Ke37 (a) and Jurkat (b) cells treated treated for the last 24 hours with *Juglone* showed that the combined treatment was not more effective in inducing cell death than single *Juglone* treatments (left panels, relative cell survival analysis) and in increasing GRP78/Bip expression and in decreasing IRE1α expression (right panels, western blot analysis). Anti–β-actin was used as a loading control.  For left panels (a) and (b) results are shown as the means average deviations of at least three separate experiments and p-values were calculated using Student’s T-test (i.e. ns, not significant). c) Control or anti-GRP78/Bip (Bip) immunoprecipates from TALL-1 (left panels), Ke37 (middle panels) and Jurkat (right panels) cells were subjected to western blot and probed with anti-N1 antibody to analyse the endogenous GRP78/Bip-Notch1 interaction. The blot with anti-GRP78/Bip antibody was used to detect the GRP78/Bip immunoprecipitated protein levels. The input lanes show 5% of total lysate.  All data are representative of at least three independent experiments, each in triplicate. *non specific band?. d) Upper panel: summary of the Notch1-IRE1α gene expression levels correlation obtained by an *in silico* analysis from two T-ALL dataset (TARGET ALL Expansion Phase 2 and GSE42328). Lower panels: representative graphs showing correlation between Notch1 and IRE1α gene expression levels from (left) TARGET ALL Expansion Phase 2 data set in a cohort of 264 T-ALL patients and (right) GSE42328 dataset in a cohort of 53 T-ALL patients. In both graphs, each dot corresponds to one patient and the expression value of Notch1 (X axis) and IRE1α (Y-axis) is given in log2 scale after normalizing data with justRMA algorithm normalization. The index Pearson R indicated expresses the linear relation between paired samples and p-values were calculated using Student’s T-test, as described in the Material and Methods Section.

**Supplementary Figure S11. Relative cell count of T-ALLs after *Juglone* treatment**

a) Notch3-positive (TALL-1, Molt3, DND41, SIL-ALL, P12-I) and Notch3-negative/low (Jurkat, KOPKT1 and Ke37) T-ALLs cells are treated with 2.5μM of *Juglone* and relative cell count was evaluated. Results are shown as the means average deviations of at least three separate experiments and p-values were calculated using Student’s T-test (i.e., *P≤0.05; **P≤0.01; ***P≤0.001).

**Supplementary Table S1. Primers sequences used for mRNA analysis of the indicated gene expression levels**
