# Supplementary material for: Notch3 contributes to T-cell leukemia growth via regulation of the unfolded protein response
Source: Oncogenesis. 2020 Oct 18;9(10):93. doi: 10.1038/s41389-020-00279-7 (PMC7569087; doi:10.1038/s41389-020-00279-7)
Supplement: Supplementary file 3 — Supplementary Materials and Methods [file 41389_2020_279_MOESM3_ESM.docx]

**Supplementary Materials and Methods**

**Flow cytometric analysis of Notch3 extracellular staining.** Cells were incubated with human Notch3 antibody (R&D Systems, Minneapolis, MN, USA, Cat#MAB1559, clone #603532) or monoclonal mouse IgG1 isotype control (R&D Systems, Cat#MAB002, clone #11711) used as negative control, as previously described (Franciosa G et al, Oncogene 2016, ref#36 of the manuscript). Then, samples were analysed on a FACS-Calibur with CellQuest software (BD-Biosciences, San Jose, CA, USA).

***In silico* analysis of T-ALL cell lines and patients deposited data**

Bone marrow lymphoblast samples from 53 T-ALL patients (Van Vlierberghe P et al, Blood 2013, ref #1 of the manuscript) and 244 bone marrow and 20 peripheral blood samples from 264 T-ALL patients (Liu Y et al, Nature Genetics 2017, ref #32 of the manuscript) were analysed for the correlation between Notch3-IRE1α and Notch1-IRE1α.

Data for 53 T-ALL patients were downloaded from R2: Genomics Analysis and Visualization Platform (http://r2.amc.nl). The expression values of Notch3 and IRE1α were filtered in each analysis utilizing the expression probe sets ILMN_1658926 and ILMN_1698404 representing Notch3 and IRE1α, respectively. The expression values for the Notch1 and IRE1α correlation were filtered using the expression probe sets ILMN_1729161 representing Notch1. The expression value of Notch3, Notch1 and IRE1α is given in log_2_ scale after normalizing data with the MAS5.0 algorithm.

Data for 264 T-ALL patients were downloaded from TARGET DATA MATRIX (<https://ocg.cancer.gov/programs/target/data-matrix>), selecting TARGET ALL Expansion Phase 2 mRNA-seq normalized data and the expression value of Notch3, Notch1 and IRE1α is given in log_2_ scale.

The index Pearson r expresses the linear relation between paired samples and was calculated with the use of GraphPad 6 (La Jolla, California, USA). P-values were calculated using Student’s T-test.

**Animal studies.** All animal experiments were approved by local ethic authorities and conducted in accordance with Italian Governing Law (D.lgs 26/2014; Prot. no. 03/2013). Animals were housed in the Institute’s Animal Care Facilities, which meet international standards and were checked regularly by a certified veterinarian responsible for health monitoring, animal welfare supervision and revision of experimental protocols and procedures.

For TALL-1 xenograft models, all the studies were conducted by using TALL-1_luc cells, generated by lentivirus infection with pLENTI-CMV-Puro-LUC (Addgene, Watertown, MA,  USA, Cat#17477) in order to track leukemia progression in vivo by optical imaging, according to previous data (Bernasconi-Elias P et al, Oncogene 2016, ref #4 of the manuscript).

To assess in vivo activity of Juglone (Sigma-Aldrich, Saint Louis, MO, USA, Cat#H47003) on Notch3 protein expression, 10*10^6^ TALL-1_luc cells were resuspended in an equal volume of MEM medium and Matrigel (BD Biosciences, Heidelberg, Germany) and injected subcutaneously at the both posterior flanks of 6-week-old female NOD/SCID/gamma (NSG) mice (Charles River Laboratories, Lecco, Italy). Tumor growth was monitored weekly by caliper measurements and mice were weighed frequently to determine treatment-induced toxicity. Once tumors reached between 300 and 500mm^3^ (after 21 days), mice were randomly assigned to receive a double intravenous 1mg/kg dose of Juglone or vehicle (Ethanol and physiological solution) for 48 hours plus 48 hours (n = 6 CTR, n = 6 Juglone-treated). At the end of the treatment, all the masses were excised and a portion of tumor was fixed in formalin and analyzed by IHC. To assess in vivo cytotoxic activity of Juglone, TALL-1_luc cells were intravenously injected (i.v.) in 6-week-old female NSG mice at 10*10^6^ cells/mouse (n=6 mice per group). On day 16, tumor-bearing animals were randomly assigned to received intraperitoneal doses of either vehicle (Ethanol and physiological solution) or Juglone (1 mg/kg), 3 days per week until a maximum of 3 weeks. For bioluminescence imaging performed at different times before killing, mice were anaesthetized, intraperitoneally injected with RediJect D-Luciferin Bioluminescence Substrate (PerkinElmer, Whaltam, MA, USA, Cat#770504), 150mg per Kg body weight, and scanned with IVIS Lumina III In Vivo Imaging System (Caliper Life Science, Waltham, MA, USA).) after 10 minutes. Animals were imaged with an exposure time of 30 seconds. Both luminescence and image data were analyzed using Living Image software (Caliper Life Science). Total flux was calculated and expressed as photons per second. No mice were excluded during the experiments.
