# Supplementary figures and images for: Notch3 contributes to T-cell leukemia growth via regulation of the unfolded protein response
Source: Oncogenesis. 2020 Oct 18;9(10):93. doi: 10.1038/s41389-020-00279-7 (PMC7569087; doi:10.1038/s41389-020-00279-7)

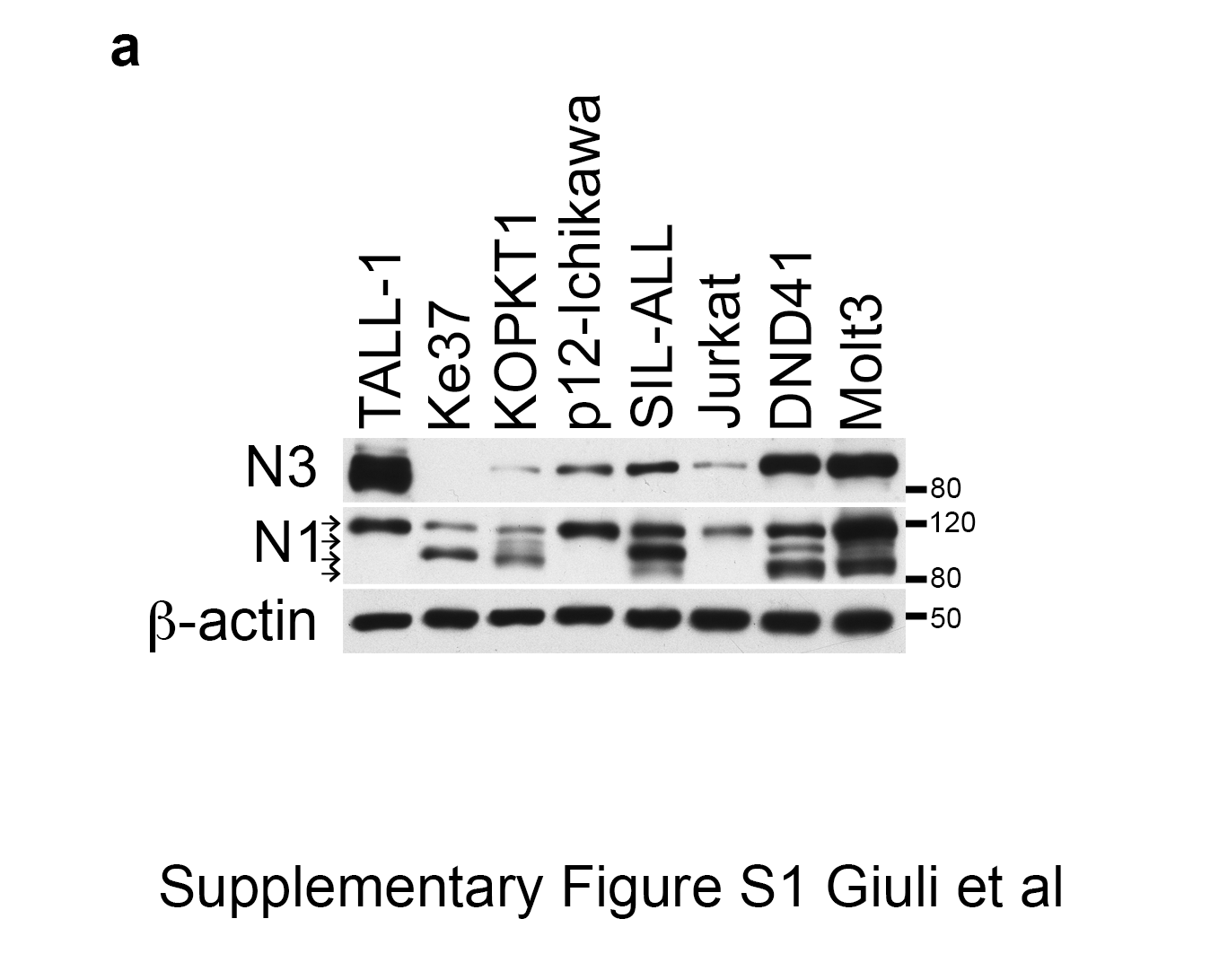

Supplement: Supplementary file 4 — Supplementary Figure S1 [file 41389_2020_279_MOESM4_ESM.tif]

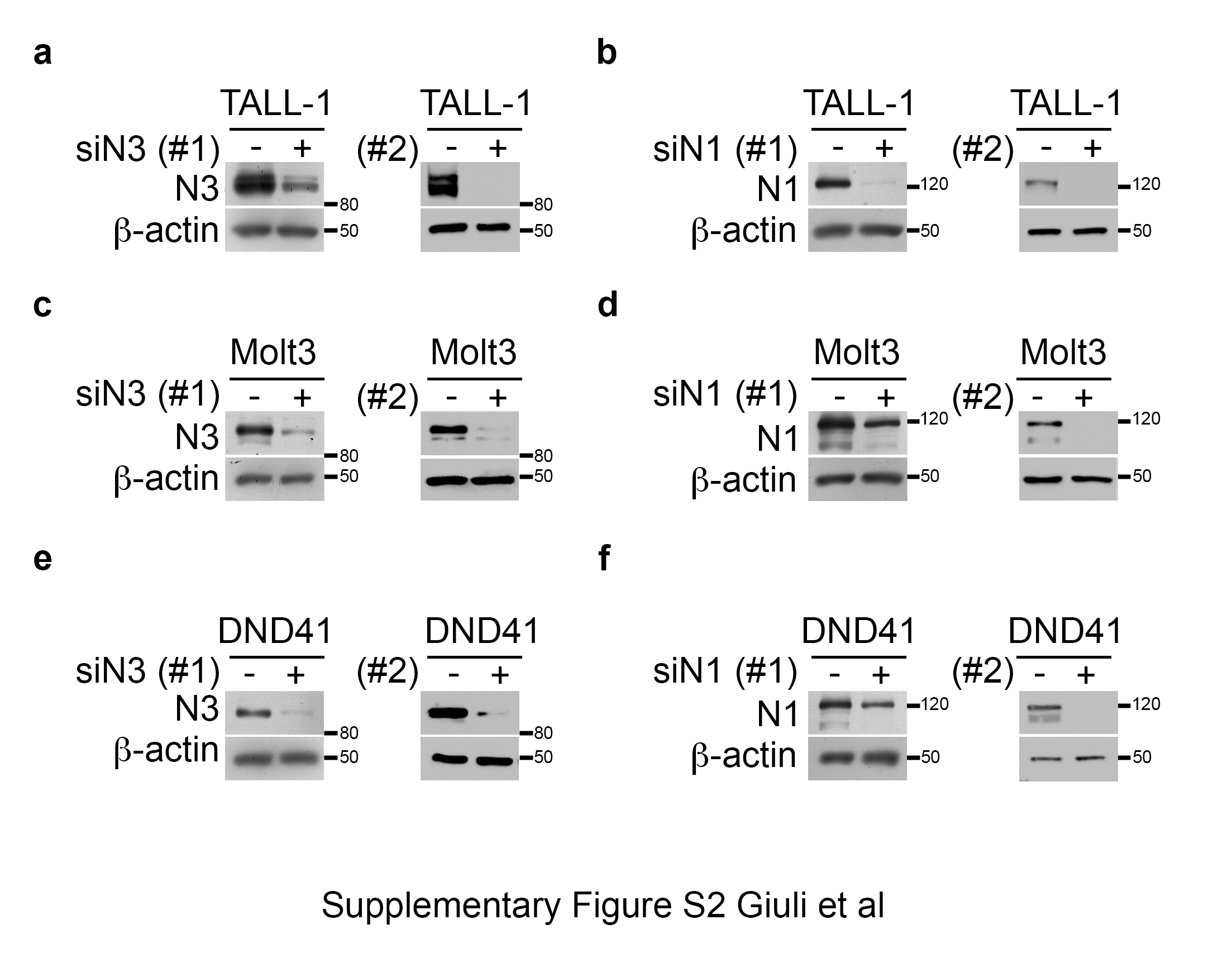

Supplement: Supplementary file 5 — Supplementary Figure S2 [file 41389_2020_279_MOESM5_ESM.tif]

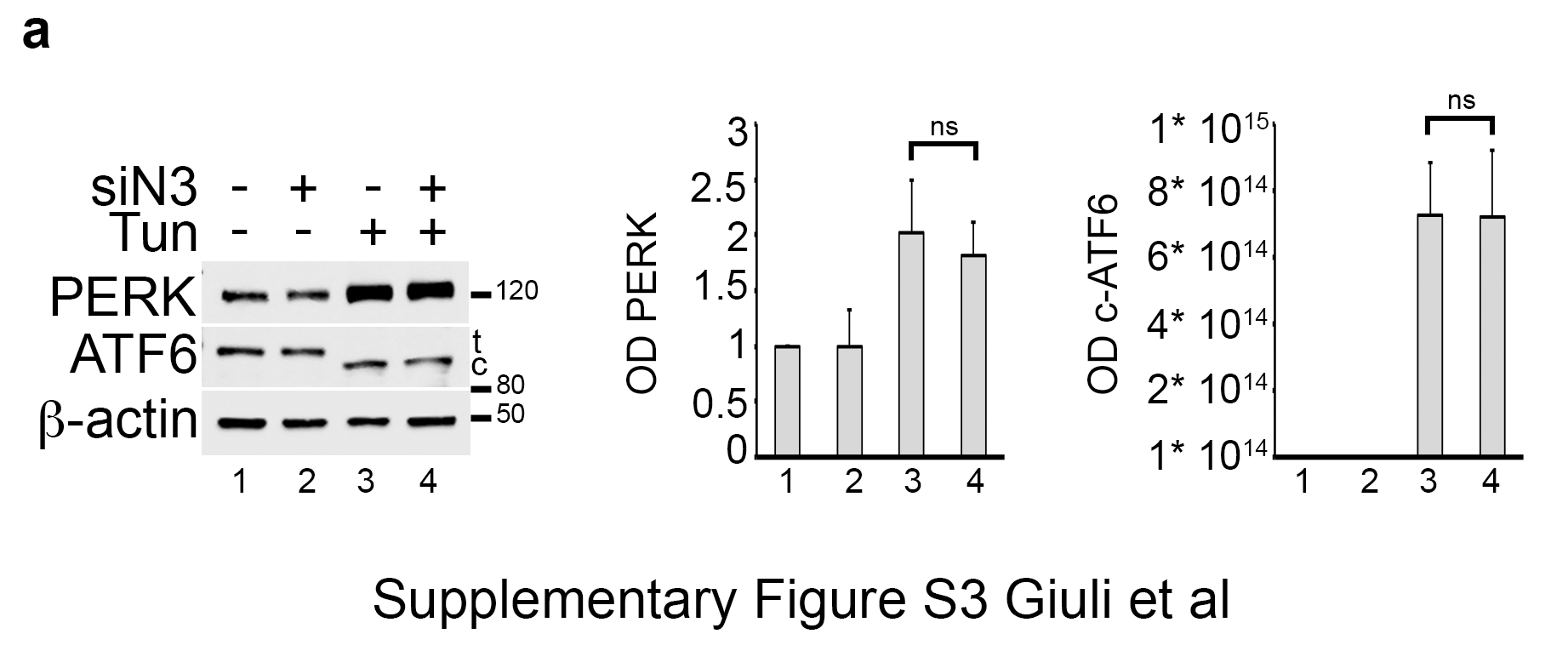

Supplement: Supplementary file 6 — Supplementary Figure S3 [file 41389_2020_279_MOESM6_ESM.tif]

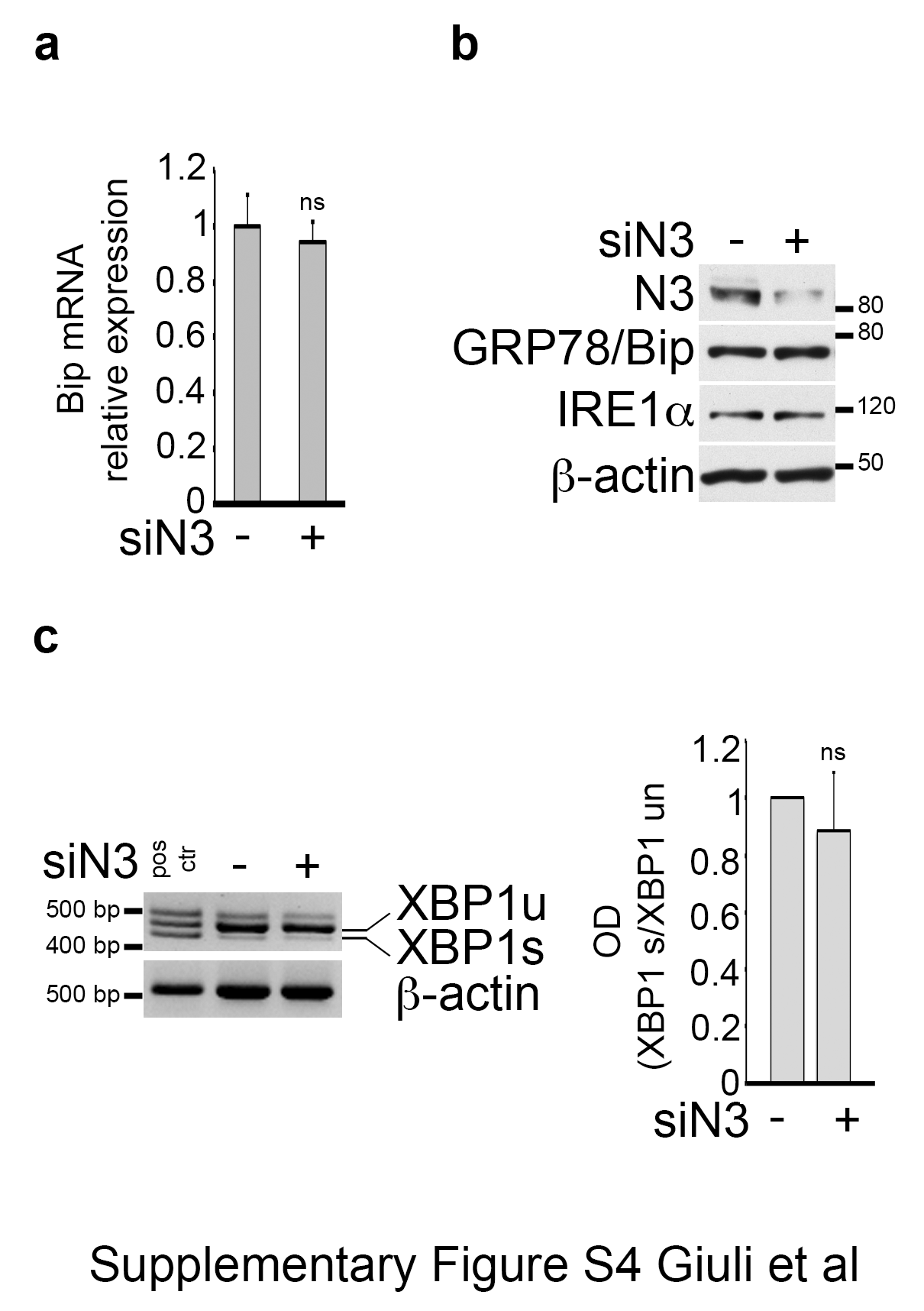

Supplement: Supplementary file 7 — Supplementary Figure S4 [file 41389_2020_279_MOESM7_ESM.tif]

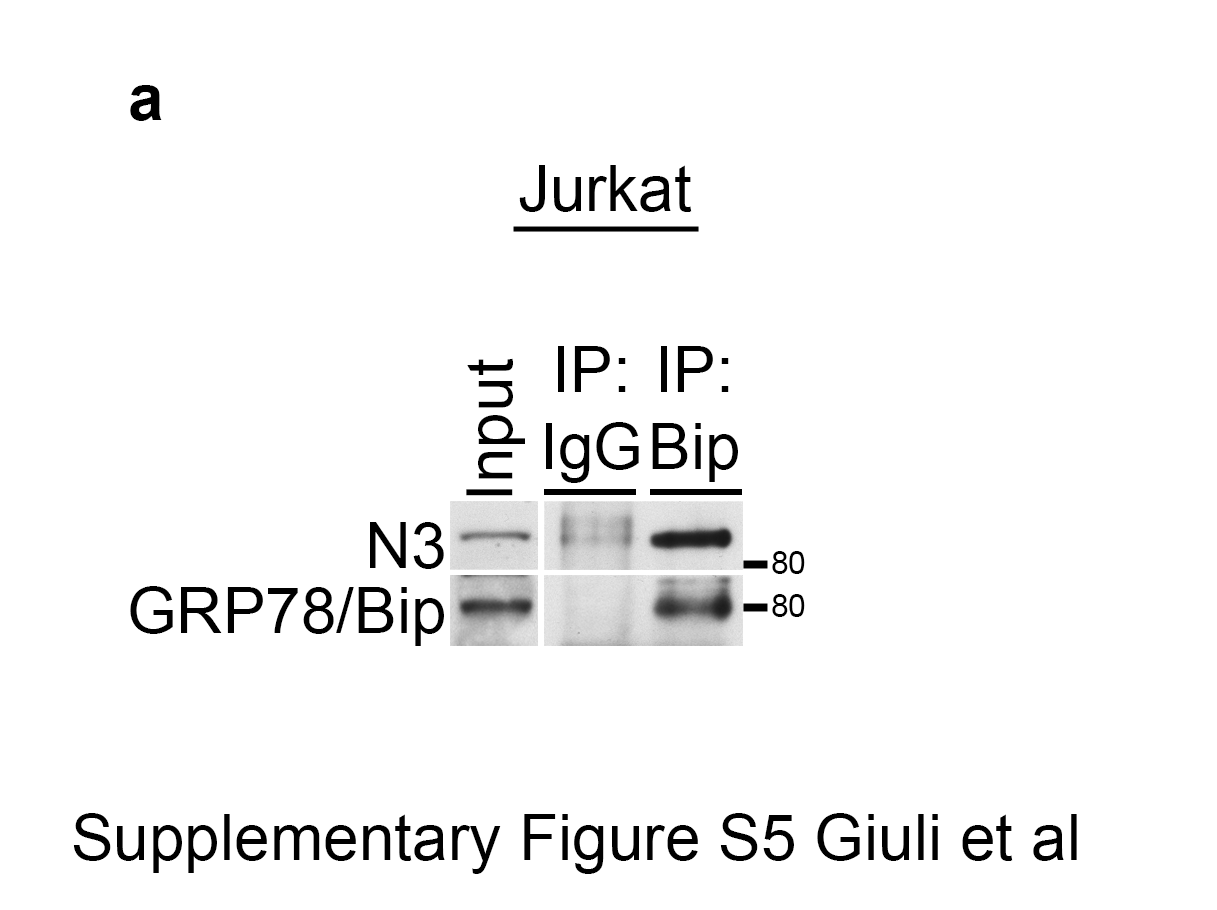

Supplement: Supplementary file 8 — Supplementary Figure S5 [file 41389_2020_279_MOESM8_ESM.tif]

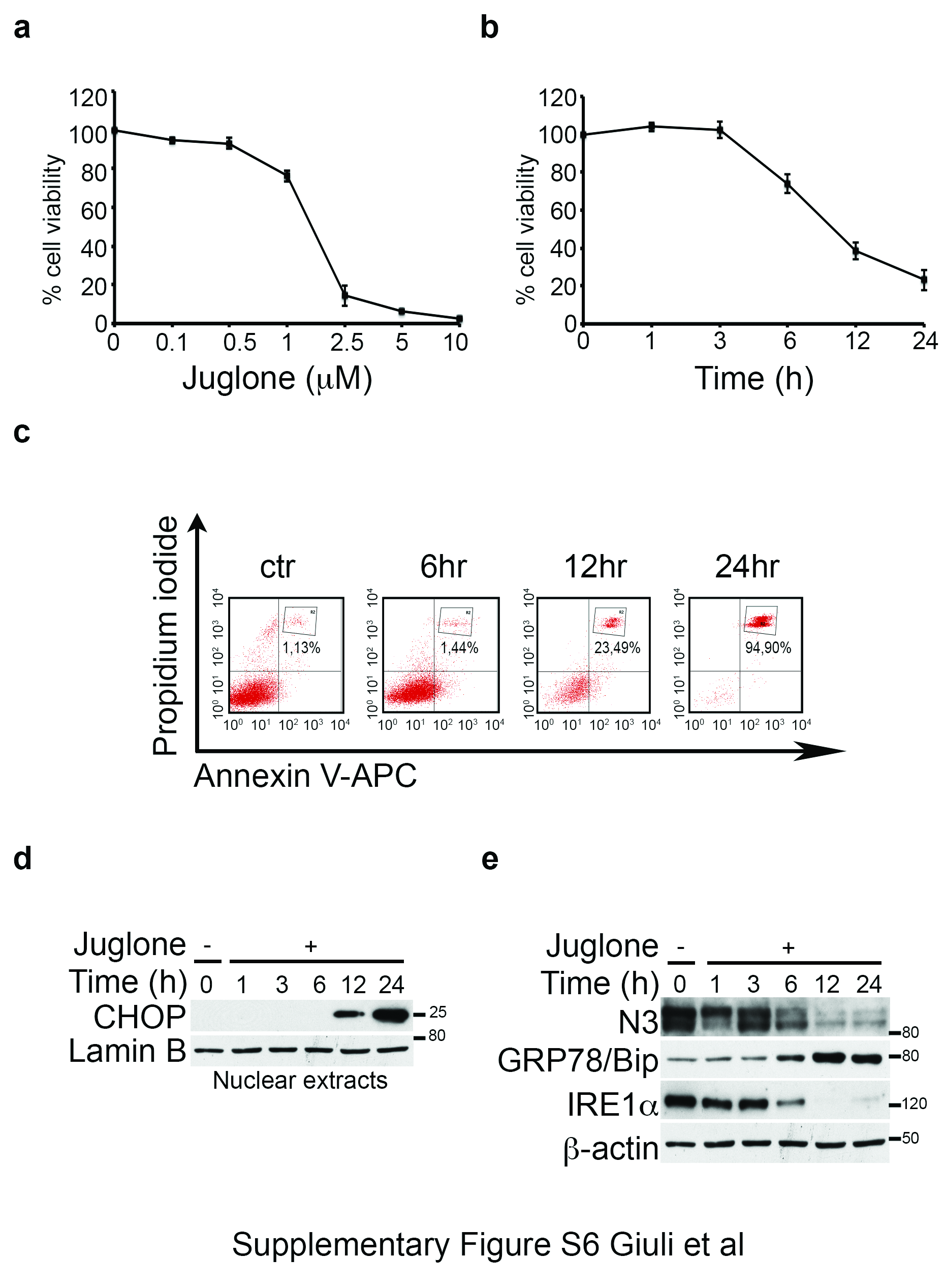

Supplement: Supplementary file 9 — Supplementary Figure S6 [file 41389_2020_279_MOESM9_ESM.tif]

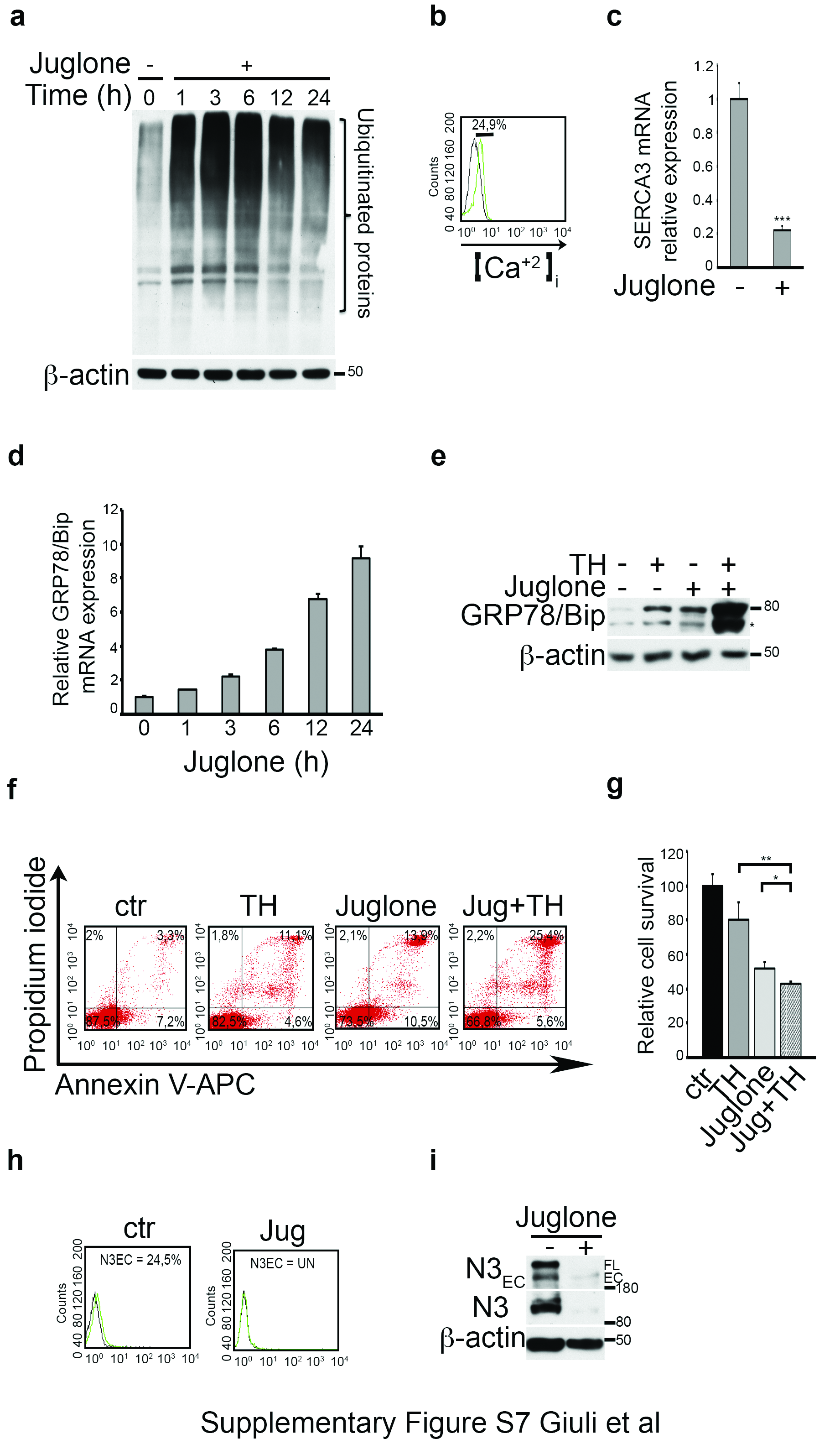

Supplement: Supplementary file 10 — Supplementary Figure S7 [file 41389_2020_279_MOESM10_ESM.tif]

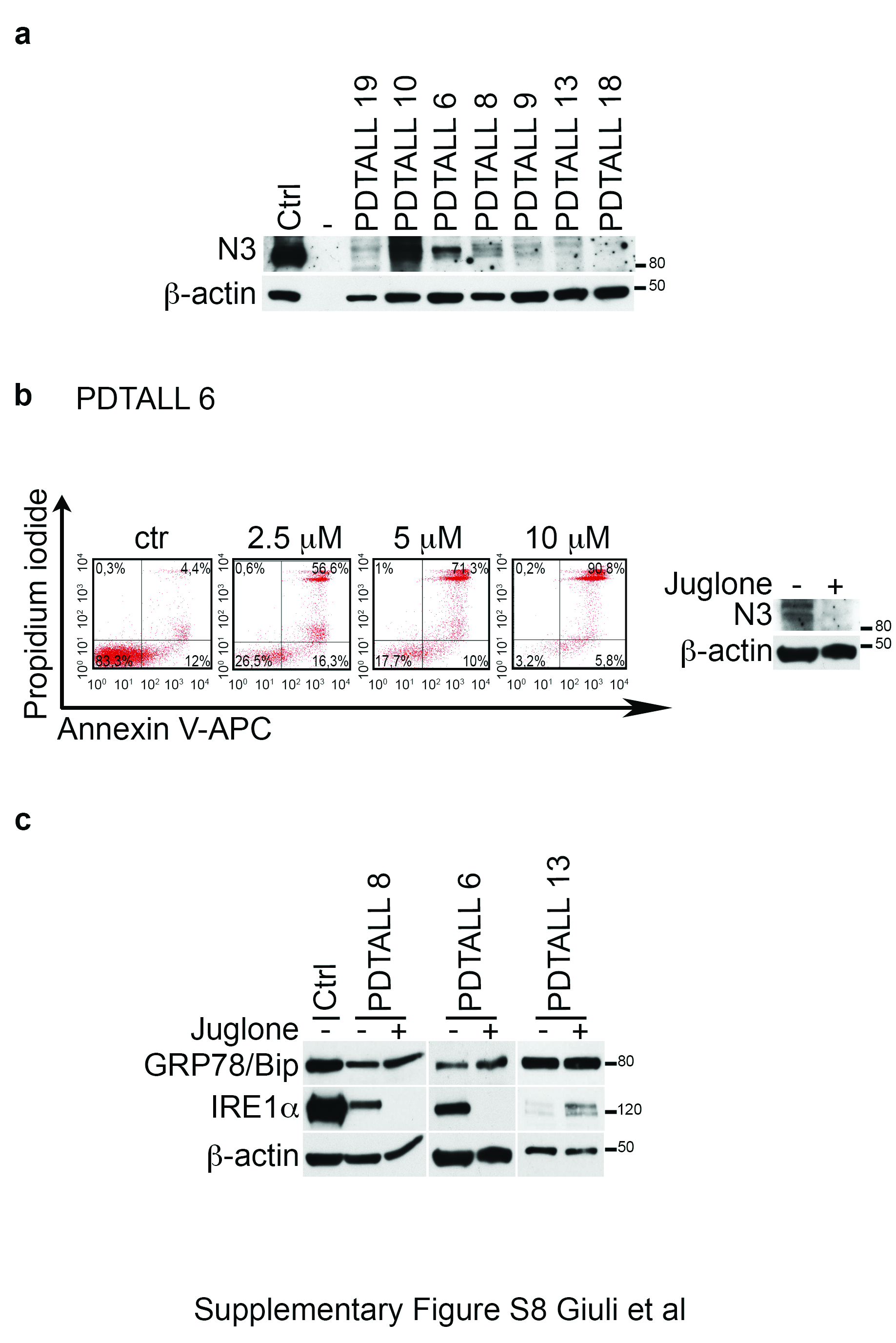

Supplement: Supplementary file 11 — Supplementary Figure S8 [file 41389_2020_279_MOESM11_ESM.tif]

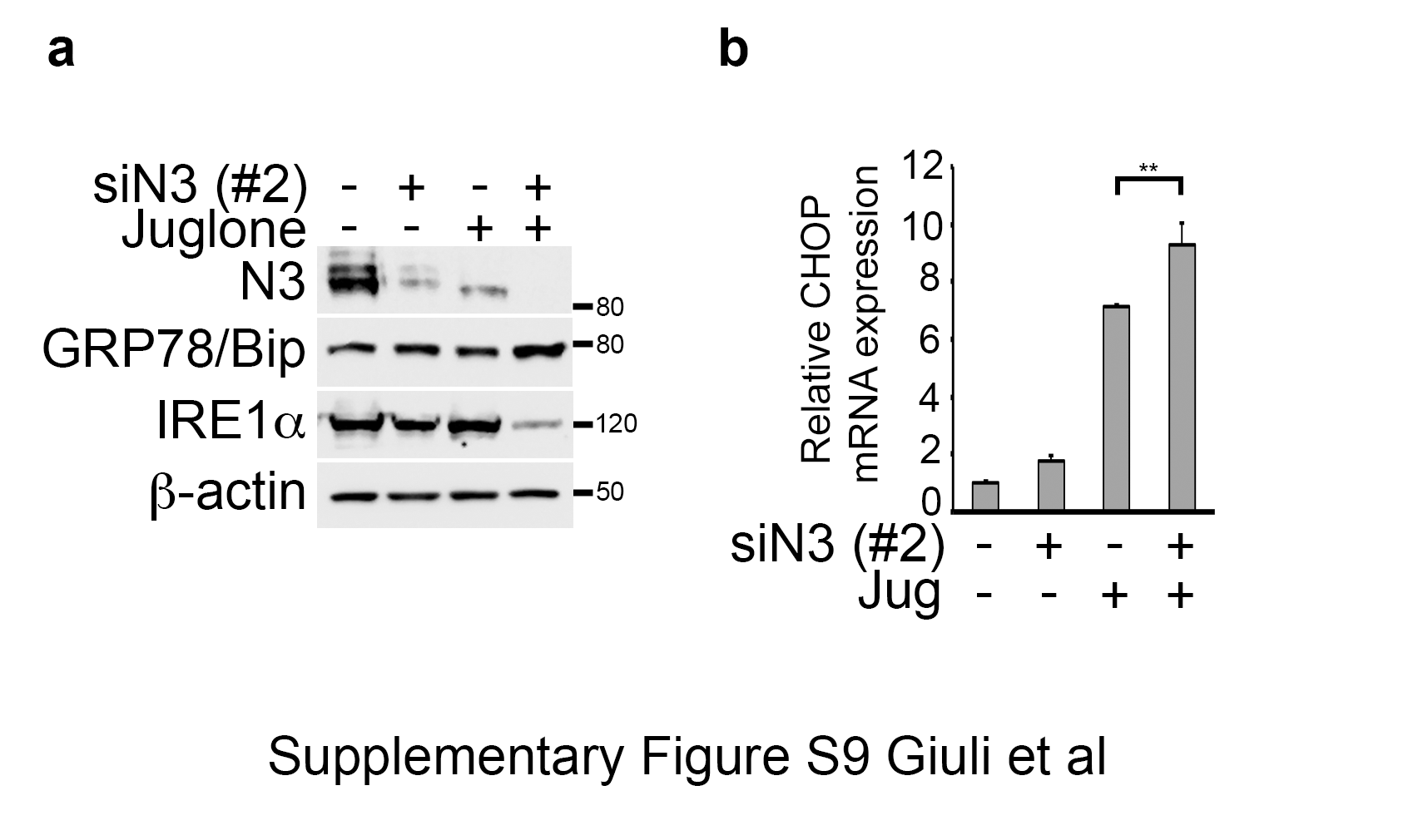

Supplement: Supplementary file 12 — Supplementary Figure S9 [file 41389_2020_279_MOESM12_ESM.tif]

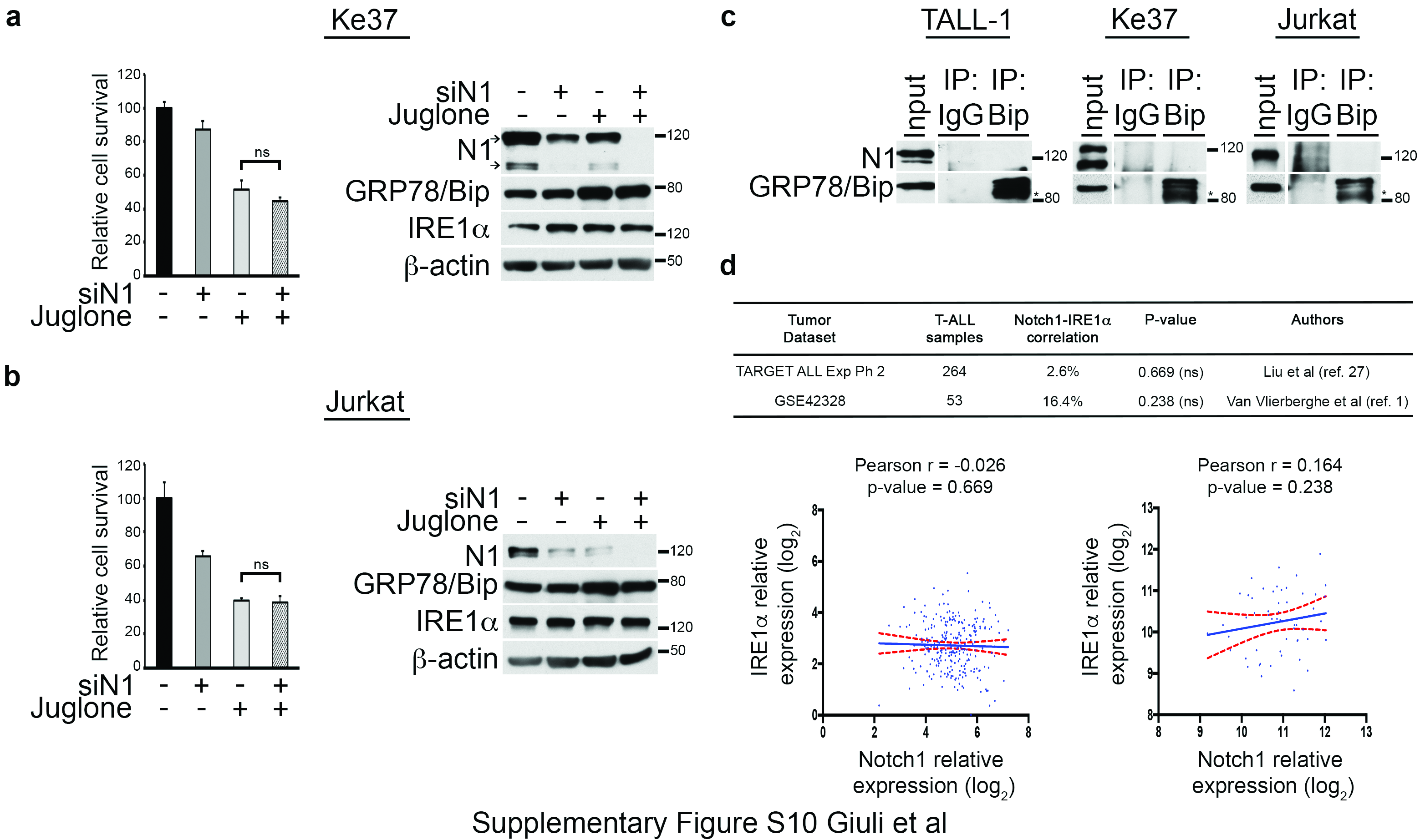

Supplement: Supplementary file 13 — Supplementary Figure S10 [file 41389_2020_279_MOESM13_ESM.tif]

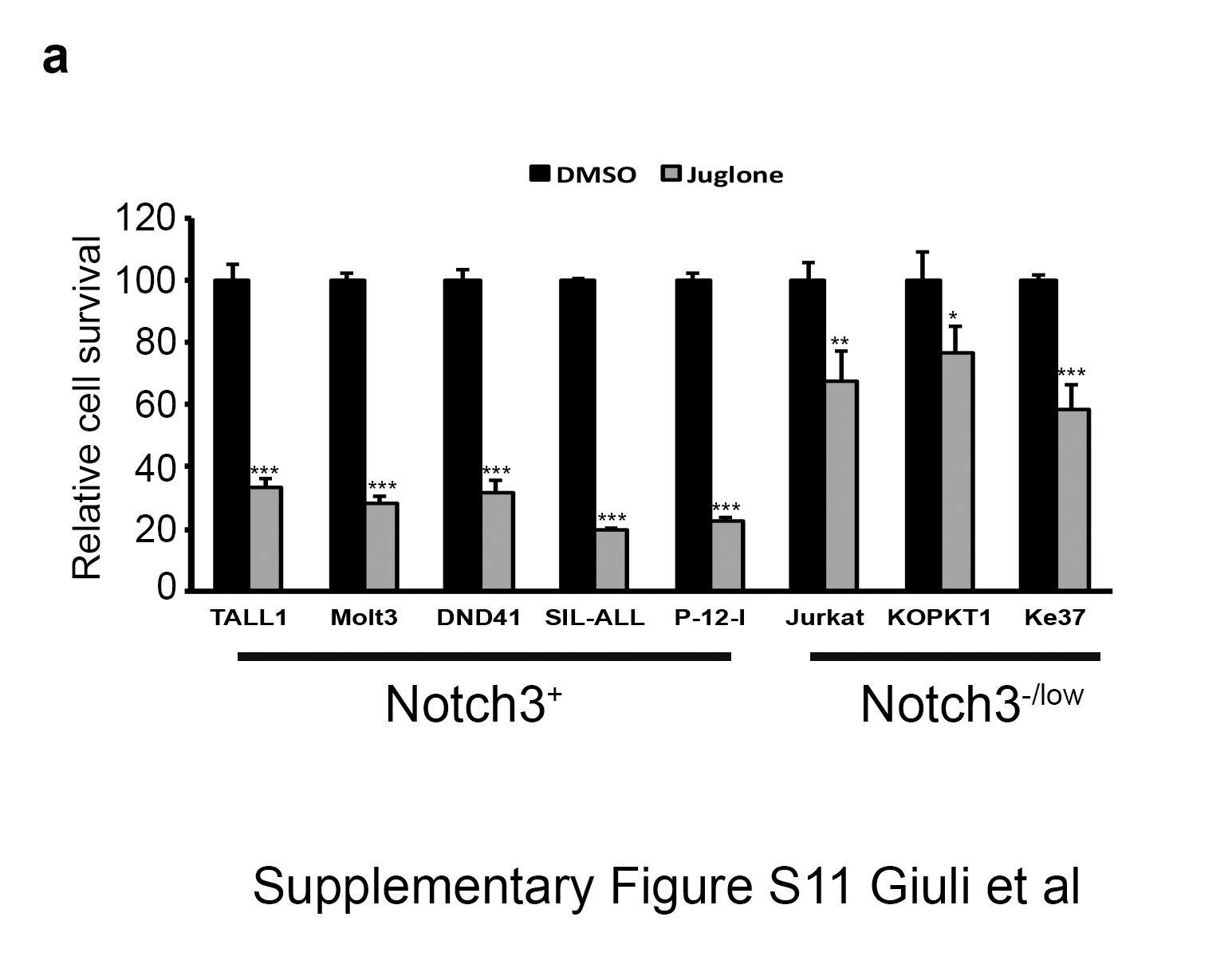

Supplement: Supplementary file 14 — Supplementary Figure S11 [file 41389_2020_279_MOESM14_ESM.tif]
